# A Circular and Tacticity‐Independent Crystalline Mono‐Substituted Nylon‐6 Platform: Unexpected Large Positional Effects on Crystallizability and Performance

**DOI:** 10.1002/anie.9256032

**Published:** 2026-05-02

**Authors:** Jun‐Jie Tian, Jiyun Nam, Lili Wang, Andrea L. Baer, Ruirui Li, Maëlle T. Gace, Wei‐Feng Zheng, Clarissa Lincoln, Nicholas A. Rorrer, Eugene Y.‐X. Chen

**Affiliations:** ^1^ Department of Chemistry Colorado State University Fort Collins USA; ^2^ Renewable Resources and Enabling Sciences Center and BOTTLE Consortium National Laboratory of the Rockies Golden USA

**Keywords:** chemical recycling, lactam, substituted nylon 6, tacticity‐independent crystallinity

## Abstract

Seeking recyclable, more sustainable alternatives to nylon 6 has drawn much attention, but achieving its variants with both crystallinity and enhanced recyclability still remains a challenge. Here, by utilizing bio‐derivable mono‐substituted racemic lactam monomers, we reveal surprisingly large effects of methyl substitution positions on the nylon‐6 backbone on crystallizability, thermomechanical performance, and recyclability of the resulting atactic nylon‐6 variants. While γ‐methyl substitution gives an amorphous nylon, all other four methyl‐substitution positions (α, β, δ, and ε) afford, unexpectedly, crystalline nylons with melting temperatures ranging from 145°C to 200°C and tunable mechanical performance from being stiff and strong (α, ε) to ductile (β, δ). Investigations reveal that the crystallinity of atactic nylons arises independently of stereoregularity, which is driven by the amide backbone with robust hydrogen‐bonding interactions and countered by the chain flexibility regulated by the substitution position. These nylons can be chemically recycled back to their parent monomers with high isolated yields up to 92%, enabling a circular, tacticity‐independent crystalline nylon platform.

## Graphical abstract

Methyl substitution positions on the nylon‐6 backbone markedly affect crystallizability, thermomechanical properties, and recyclability of the resulting atactic nylon‐6 variants. While γ‐methyl substitution gives an amorphous nylon, all other four methyl‐substitution positions (α, β, δ, and ε) afford, unexpectedly, crystalline nylons, showing tacticity‐independent crystallinity.

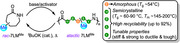

## 1 Introduction

Commercial plastics are predominantly semi‐crystalline polymers whose crystalline domains often provide high mechanical strength and high resistance to temperature, deformation, gas permeation, and chemicals [1, 2, 3]. For polymers bearing stereogenic centers, the degree of stereoregularity or tacticity is the primary determinant of crystallinity: stereoirregular, atactic chains are typically non‐crystallizable and thus amorphous, whereas stereoregular (isotactic, syndiotactic, or stereoblock) chains can pack efficiently into ordered crystalline domains to display semi‐crystallinity [1, 2, 3, 4]. Polypropylene (PP) exemplifies this principle: atactic PP is amorphous, whereas isotactic and syndiotactic PP are semicrystalline, highlighting how stereochemistry dictates morphology and material properties [5]. Consequently, the synthesis of such semicrystalline polymers requires elaborate stereoselective catalysts [6, 7, 8] or the use of synthetically challenging chiral monomers to achieve high polymer stereoregularity [9, 10, 11, 12]. However, some polymers defy this paradigm where their crystallinity is independent of tacticity, such that even atactic forms remain semicrystalline. Notable examples include polar‐group‐functionalized vinyl polymers such as polyacrylonitrile [13], poly(vinyl chloride) [14, 15], and poly(vinyl alcohol) [16, 17], as well as hydrogenated polynorbornenes [18] and poly(thio)esters [19, 20, 21]. While the fundamental reasons why such atactic polymers can crystallize vary (e.g., derived by hydrogen bonding, dipole interaction, conformational flexibility, or steric induction) and merit further studies, these tacticity‐independent crystalline materials offer practical advantages, as their semicrystalline morphology can be accessed for performance using readily available, inexpensive racemic monomers and non‐stereoselective catalysts.

**FIGURE 1:**
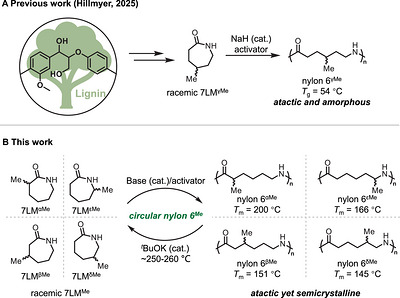
Selected examples of mono‐substituted nylon‐6 variants. (A) Previously reported atactic and amorphous γ‐methyl‐substituted nylon. (B) This work: atactic yet semicrystalline methyl‐substituted nylon‐6 variants with full chemical circularity.

Nylon 6 is one of the most widely used aliphatic polyamides (nylons) and exhibits extensive applications in textiles and engineering plastics [22, 23, 24, 25, 26]. Its semicrystalline structure imparts a combination of high melting temperature (*T*_m_), mechanical strength and stiffness, as well as thermal and chemical resistance. Industrially, nylon 6 is produced via the anionic ring‐opening polymerization (AROP) of petroleum‐derived seven‐membered ε‐caprolactam (7LM). In recent years, renewable biomass feedstocks have emerged as more sustainable alternatives for the development of nylon monomers (i.e., biobased lactams) that could replace petroleum‐based lactams, including 7LM, aiming to advance the sustainability of nylon materials [27, 28, 29, 30, 31, 32, 33, 34]. In parallel, structural modification through introducing substituents onto the polyamide backbone has been recognized as an effective strategy to tailor the crystallinity, toughness, transparency, and recyclability [27, 28, 29, 30, 31, 32, 33, 34, 35, 36, 37, 38, 39, 40]. Notably, Hillmyer and co‐workers demonstrated that γ‐methyl nylon 6 (nylon 6^γMe^) derived from lignocellulosic resources exhibits high stiffness, strength, toughness, and optical clarity under ambient conditions (Figure 1A) [33, 34]. Nevertheless, the resulting nylon 6 variant is amorphous as lignin‐derived mono‐substituted lactam monomers contain a stereogenic center and are obtained as racemates. When polymerized via AROP with achiral bases (e.g., NaH or *^t^* BuOK), these lactams led to atactic, amorphous nylons [33, 34]. The absence of crystalline domains in the resulting nylons exhibits poor thermal resistance (no *T*_m_, coupled with a low glass‐transition temperature (*T*_g_) ∼ 54°C), diminished chemical stability, and limited dimensional integrity, constraining their utility as high‐performance fibers that nylons are known for.

Herein, we report that, in contrast to the reported atactic, amorphous nylon 6^γMe^ [34, 35, 36], α‐, β‐, δ‐, and ε‐methyl 7LMs unexpectedly afford a series of semicrystalline methyl‐substituted nylon 6 variants, despite being atactic (Figure 1B). Studies on these surprising observations reveal that methyl‐substitution positions along the nylon‐6 backbone can override the conventional dependence of crystallinity on tacticity. Through systematic investigations into how the substitution site influences polymer crystallinity, and thermal and mechanical properties, we gain fundamental insights into structure–property relationships in mono‐substituted nylons.

## 2 Results and Discussion

### 2.1 Substitution Site–Reactivity Relationship in AROP of 7LM^Me^

Methyl‐substituted ε‐caprolactams (7LM^Me^) at α‐, β‐, δ‐, and ε‐positions were synthesized from 2‐ and 3‐methylcyclohexan‐1‐one, which could be derived from *para*‐ and *meta*‐cresol lignin sources [41] via the Beckmann rearrangement. Their AROP reactions were subsequently examined in detail, with the results summarized in Table 1.

**TABLE 1:** Polymerization of methyl‐substituted ε‐caprolactams.^a^

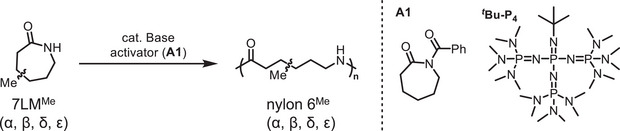								
Entry	Monomer	[M]/[B]/[A]	T (°C)	Base	Solvent	Yield^b^ (%)	*M*_n_ ^c^ (kDa)	*Ð* ^c^
1	7LM^αMe^	100/1/1	60	*^t^* Bu‐P_4_	DMAc^e^	59	7.13	1.05
2	7LM^αMe^	100/1/1	80	*^t^* Bu‐P_4_	DMAc^e^	78	6.90	1.49
3	7LM^αMe^	100/1/1	100	*^t^* Bu‐P_4_	Neat	91	13.7	2.79
4^d^	7LM^αMe^	500/1/1	100	*^t^* Bu‐P_4_	Neat	90	38.6	2.36
5^d^	7LM^αMe^	750/1/1	100	*^t^* Bu‐P_4_	Neat	91	87.0	2.14
6^d^	7LM^αMe^	1000/2/1	100	*^t^* Bu‐P_4_	Neat	77	56.5	2.28
7	7LM^βMe^	100/1/1	100	*^t^* Bu‐P_4_	Neat	90	14.6	1.77
8^d^	7LM^βMe^	500/1/1	100	*^t^* Bu‐P_4_	Neat	85	36.1	2.54
9^d^	7LM^βMe^	1000/1/1	100	*^t^* Bu‐P_4_	Neat	85	77.1	1.64
10	7LM^δMe^	100/1/1	80	*^t^* Bu‐P_4_	Neat	85	21.0	1.76
11^d^	7LM^δMe^	500/1/1	80	*^t^* Bu‐P_4_	Neat	74	55.7	2.18
12	7LM^εMe^	100/1/1	140	NaH	Neat	83	34.4	3.26
13^d^	7LM^εMe^	500/1/1	140	NaH	Neat	81	103	2.55

^a^ Conditions: monomer (M) = 2 mmol, base (B) = *^t^* Bu‐P_4_ or NaH (60 wt%), activator (A) = *N*‐benzoyl‐7LM (**A1**), reaction time = 12 h.

^b^ Isolated yield.

^c^ Number‐average molar mass (*M*_n_) and dispersity (*Ð* = *M*_w_ */M*_n_) determined via size exclusion chromatography (SEC) coupled with a 3‐angle light scattering detector.

^d^ 1.27 g, 10 mmol monomer, 24 h.

^e^ 2 M.

For 7LM^αMe^, the AROP proceeded efficiently with superbase *^t^* Bu‐P_4_ as catalyst and *N*‐benzoyl‐7LM (**A1**) as activator (which is typically required for AROP of 7LMs), with the polymerization behavior depending on temperature, solvent, and monomer concentration. Specifically, in *N,N*‐dimethylacetamide (DMAc), nylon 6^αMe^ was obtained in 59% (60°C) and 78% (80°C) yield in 12 h, but the nylons had low a number‐average molar mass (*M*_n_) values of ∼7 kDa and dispersity (*Ð*) between 1.05 and 1.49 with a [monomer]/[base]/[activator] ratio of 100:1:1 (Entries 1 and 2). Switching to solventless conditions at 100°C markedly increased both yield (91%) and molar mass (*M*_n_ = 13.7 kDa, Entry 3). Further increasing the ratio to 500/1/1, 750/1/1, and 1000/2/1 and extending the reaction time to 24 h afforded nylon 6^αMe^ with *M*_n_ = 38.6–87.0 kDa (*Ð* = 2.36–2.14) in 77–91% yield (Entries 4–6).

Under similar conditions, *^t^* Bu‐P_4_ also enabled the AROP of 7LM^βMe^ and 7LM^δMe^, yielding nylon 6^βMe^ and nylon 6^δMe^ in yields ranging from 74% to 90% and *M*_n_ values from 14.6 kDa (*Ð* = 1.77) to 77.1 kDa (*Ð* = 1.64), depending on the substitution site and the ratio (Entries 7–11). In contrast, the sterically encumbered 7LM^εMe^ was inreactive with *^t^* Bu‐P_4_, as it is too bulky to abstract the proton from the sterically protected amide N–H bond in 7LM^εMe^ to generate an active anionic species in the initiation step. Accordingly, switching the base to unhindered NaH at 140°C successfully mediated the AROP, affording nylon 6^εMe^ with *M*_n_ up to 103 kDa (*Ð* = 2.55) (Entries 12 and 13).

### 2.2 Effects of Substitution Positions on Material Properties and Crystallinity

**FIGURE 2:**
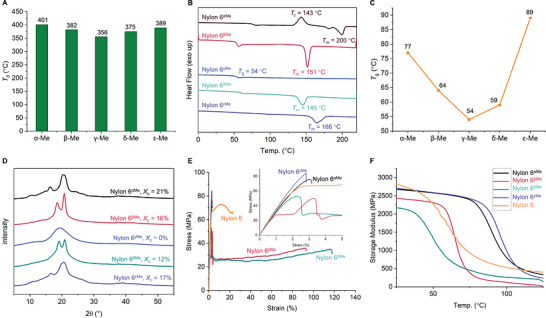
Thermal properties, degree of crystallinity, and mechanical performance of methyl nylon‐6 variants. (A) TGA‐derived *T*_d_ values (10°C/min). (B) DSC thermograms (10°C/min), second heating DSC scan curves for nylon 6^αMe^ and nylon 6^γMe^, and first heating DSC scan curves for nylon 6^βMe^, nylon 6^δMe^, and nylon 6^εMe^. (C) *T*_g_ values from second heating DSC scan curves; (D) WAXS profiles and crystallinity degrees. (E) Overlays of representative stress–strain curves. (F) DMA (tension film mode) thermomechanical profiles. Storage modulus (*E′*) values shown were taken at 25°C.

With nylon 6^αMe^, nylon 6^βMe^, nylon 6^δMe^, and nylon 6^εMe^ in hand, we next examined their thermal properties and compared them to the reported data on commercial nylon 6 [42] and nylon 6^γMe^ [35, 36]. Thermogravimetric analysis (TGA) revealed their high onset decomposition temperatures at 5% mass loss (*T*_d_) of 401°C, 382°C, 356°C, 375°C, and 389°C, respectively (Figure 2A). Nylon 6^αMe^ exhibited a *T*_d_ comparable to that of nylon 6 (400°C), whereas the other methylated nylon‐6 variants displayed slightly lower thermal stability. Unexpectedly, differential scanning calorimetry (DSC) showed *T*_m_ endotherms at 200°C, 151°C, 145°C, and 166°C, accompanied by *T*_g_ endotherms of 77°C, 64°C, 59°C, and 89°C, for the α‐, β‐, δ‐, and ε‐methyl nylon 6, respectively (Figure 2B,C). Unlike the amorphous γ‐methyl analogue nylon 6^γMe^, these new methyl nylons are all semicrystalline, albeit with lower *T*_m_ values than nylon 6 (220°C). Wide‐angle x‐ray scattering (WAXS) further confirmed their crystallinity, displaying sharp diffraction peaks, and their crystalline degree (*χ*_c_) varied from 12% to 21%, which is lower than that of dry nylon 6 at 42% (Figure 2D). Small‐angle x‐ray scattering (SAXS) of nylon 6^αMe^ and nylon 6^εMe^ displayed peaks shifted to *q* values of 0.81 and 0.75 nm^−^^1^, respectively, indicating interlamellar spacings of approximately 8 nm (Figure S39) [43]. In contrast, both nylon 6^βMe^ and nylon 6^δMe^ exhibited only weak and broad scattering features, indicative of a loss of well‐defined lamellar ordering and consistent with their low crystallinity degree. Overall, these results indicate that incorporation of methyl groups at α‐, β‐, δ‐, or ε‐positions along the nylon 6 backbone does not abolish semicrystallinity, although it reduces both crystallinity and *T*_m_ by lowering chain packing efficiency. Their *T*_m_, *T*_g,_ and crystallinity decrease progressively from α to γ substitution and then increase again from γ to ε, indicating that the position of the methyl group notably affects the thermal properties and crystallinity of the resulting nylon‐6 variants.

Tensile tests were performed to evaluate the effect of the position of methyl substitution on the mechanical properties of the resulting methyl nylon‐6 variants (Figure 2E). When the methyl group is substituted at the α position, the corresponding nylon 6^αMe^ (38.6 kDa) exhibited a high Young's modulus (*E*) of 3.3 ± 0.2 GPa and a high yield stress (*σ*_y_) of 76.1 ± 3.8 MPa, comparable to those of commercial nylon 6 (14.0 kDa, *E =* 3.4 ± 0.1 GPa, *σ*_y_ = 72.5 ± 0.8 MPa) [27, 28, 29, 30, 31, 32]. However, nylon 6^αMe^ is more brittle, with an elongation at break (*ε*_B_) of only 3.3 ± 0.2% versus 25.9 ± 11.6% for nylon 6. Notably, nylon 6^αMe^ with a higher molar mass (*M*_n_ = 87.0 kDa) did not show improved mechanical performance compared to the lower molar mass sample (*M*_n_ = 38.6 kDa), indicating that molar mass does not significantly influence the mechanical properties of these nylon materials once a sufficient chain length is reached (Figure S40). In contrast, methyl substitution at the β and δ positions yields more ductile materials: nylon 6^βMe^ (36.1 kDa) and nylon 6^δMe^ (55.7 kDa) both display greatly increased elongations at break (*ε*_B_ = 98.3 ± 6.9% and 124 ± 6.4%, respectively) compared to nylon 6, and similar Young's modulus (*E* = 3.3 GPa) but lower yield stresses (*σ*_y_ = 49.8 ± 0.7 MPa and 52.3 ± 2.8 MPa, respectively). When the methyl group is located at the ε position, nylon 6^εMe^ (103 kDa) shows a higher modulus (*E* = 3.6 ± 0.1 GPa) and yield stress (*σ*_y_ = 81.6 ± 3.6 MPa), but it is also brittle with *ε*_B_ of only 2.8 ± 0.05%. Overall, these results reveal that α‐ or ε‐substitution stiffens the polymer by raising the modulus and yield strength at the expense of flexibility, whereas β‐ or δ‐substitution softens the material and enhances ductility while lowering the yield stress. Dynamic mechanical analysis (DMA) in the tension‐film mode was used to examine the thermomechanical behavior of the four methyl‐substituted nylons (Figure 2F). At 25°C (i.e., in the glassy state), nylon 6 displayed a storage modulus (*E′*) of 2.81 GPa. In comparison, nylon 6^αMe^ and nylon 6^εMe^ exhibited similar moduli of 2.68 and 2.71 GPa, respectively, whereas both nylon 6^βMe^ and nylon 6^δMe^ showed noticeably reduced values of 2.43 and 2.17 GPa, respectively. The *T*_g_ values, taken from the peak maxima on tan *δ* (loss modulus/storage modulus), were 97°C, 70°C, 57°C, and 103°C for nylon 6^αMe^, nylon 6^βMe^, nylon 6^δMe^, and nylon 6^εMe^, respectively (Figures S44–S47).

To evaluate the effect of methyl substitution position on the hygroscopic behavior of nylon materials, we monitored water absorption after 7 days of immersion in water. Nylon 6 showed a mass increase of 8.76 ± 0.40%, while introduction of a methyl group at the α‐position reduced the water uptake to 7.03 ± 0.11% (Figure S48), indicating enhanced hydrophobicity. In contrast, methyl substitution at more distal positions along the backbone led to increased water absorption, with β‐Me, δ‐Me, and ε‐Me samples showing mass increases to 8.9 ± 0.25%, 10.4 ± 0.35%, and 11.1 ± 0.31%, respectively. This trend suggests that the position of methyl substitution plays a critical role in governing water uptake: substitution closer to the carbonyl group suppresses water absorption, whereas substitution further along the chain progressively enhances it.

### 2.3 Tacticity Variations and Tacticity‐Independent Crystallinity

The above unexpected findings that, even in the absence of chiral catalysts to enforce stereocontrol in the AROP of racemic 7LMs, the methyl‐substituted nylon‐6 variants, except for nylon 6^γMe^ being amorphous, all exhibit a certain degree of crystallinity with a distinct *T*_m_ peak on DSC thermograms prompted us to investigate the origin of their crystallinity.

We hypothesized that this unexpected behavior could arise from two possible scenarios: (i) the AROP is stereoselective via a possible chain‐ended mechanism, in which the stereogenic center of the last‐inserted monomer dictates the enantiofacial selectivity of the next enchaining monomer despite the catalyst being achiral, leading to construction of stereoregular chains [44, 45]; (ii) the polymers are indeed stereoirregular, atactic but intrinsically crystalline such that their crystallinity is independent of tacticity. Typically, tacticity of the polymers bearing stereogenic centers is assessed by ^13^C NMR spectroscopy; however, all carbon peaks of these nylons (which are typically sensitive to how chiral centers are organized along the chain and used to access tacticity) appear as singlets in trifluoroacetic acid‐*d*_1_ or dimethyl sulfoxide‐*d*_6_ (Figures S9–S12), likely because the stereocenters between the adjacent repeating units are too spatially far away from each other to create distinct diastereomeric environments characterizable by ^13^C NMR [46].

**FIGURE 3:**
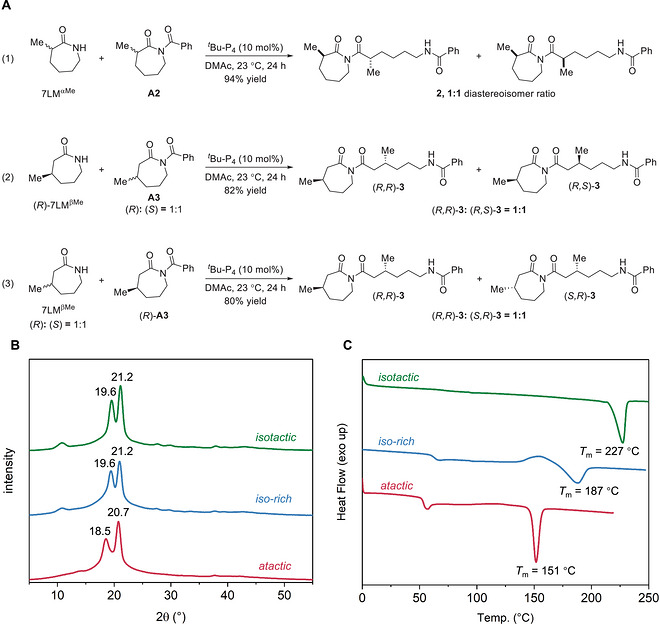
(A) Outlined scheme for control reactions specifically designed to probe the stereoselectivity of AROP. (B) WAXS profiles of isotactic, iso‐rich, and atactic nylon 6^βMe^. (C) Second heating DSC scan (10°C/min) curves for isotactic, iso‐rich nylon 6^βMe^, and first heating DSC scan (10°C/min) curves for atactic nylon 6^βMe^.

Owing to a lack of the ability to probe whether chain propagation is stereocontrolled or not by analyzing the polymer stereoregularity with ^13^C NMR, we carried out specifically designed control reactions that mimic the chain‐growth step (Figure 3A). First, racemic 7LM^αMe^ considered as the next monomer and acyl 7LM^αMe^ (**A2**) as the last inserted monomer were subjected to the reaction in the presence of *^t^* Bu‐P_4_ and DMAc (1 M) at 23°C, affording **2** in 94% yield with a 1:1 diastereomeric ratio (Figure 3A, Equation (1); Figure S5). In a second study, (+)‐pulegone was employed as a renewable chiral source to synthesize enantiopure (*R*)‐7LM^βMe^ and the corresponding acyl (*R*)‐7LM^βMe^ ((*R*)‐**A3**) [47]. Reaction of enantiopure (*R*)‐7LM^βMe^ with racemic **A3** under identical conditions produced (*R*,*R*)‐ and (*R*,*S*)‐**3** in 82% yield, also with a 1:1 ratio (Figure 3A, Equation (2); Figure S7). Likewise, coupling of racemic 7LM^βMe^ with enantiopure (*R*)‐**A3** also afforded (*R*,*R*)‐ and (*S*,*R*)‐**3** in 80% yield, still with a 1:1 ratio (Figure 3A, Equation (3); Figure S8). The results obtained from the above control reactions indicated no stereochemical preference for the addition of the next monomer toward the last inserted monomer in propagation. Therefore, the polymerization of 7LM^Me^ catalyzed by achiral bases (*^t^* Bu‐P_4_ or NaH) proceeded without stereochemical control, indicating that the resulting (α, β, δ, ε) methyl‐substituted nylon‐6 variants are predominantly atactic but intrinsically semicrystalline.

To provide direct evidence that the crystallinity is independent of tacticity, WAXS profiles were collected for isotactic (*M*_n_ = 11.8 kDa, *Đ* = 2.61), iso‐rich (*M*_n_ = 15.5 kDa, *Đ* = 1.88), and atactic nylon 6^βMe^ (*M*_n_ = 14.6 kDa, *Đ* = 1.77) (Figure 3B), which were synthesized from enantiopure (*R*)‐7LM^βMe^, enantioriched (*R*)‐7LM^βMe^:7LM^βMe^ in 1:1 ratio, and racemic 7LM^βMe^, respectively (Table S1). All the three samples exhibit sharp diffraction peaks characteristic of crystalline phases. The isotactic and iso‐rich samples show reflections at 2*θ* = 19.6° and 21.2°, whereas the atactic sample displays peaks at 18.5° and 20.7°. Although these three samples are all semicrystalline, the degree of tacticity significantly affects their thermal behavior (Figure 3C): increasing the stereoregularity leads to a higher *T*_m_, rising from 151°C for the atactic sample to 187°C for the iso‐rich sample and 227°C for the isotactic polymer.

On the basis of poly(vinyl alcohol) [17], we postulate that the strong, directional hydrogen bonds along the amide backbone provide a sufficient driving force for chain organization and ordered crystal packing, effectively overriding the configurational disorder of the side‐chain methyl groups. In contrast to the amorphous polyester analog poly(methyl‐ε‐caprolactone) [41], which lacks strong interchain hydrogen bonding, this robust hydrogen‐bonding network is able to tolerate side‐chain methyl group's spatial irregularities, enabling the atactic methyl‐substituted nylons to effectively crystallize.

Thermodynamically, we reason that crystallization ongoing from the polymer melt or solution to the crystalline state in these tacticity‐variable nylon materials reflects a balance or tradeoff between the favorable enthalpy gained from more dense, robust hydrogen bonding and enhanced interchain interactions due to chain packing (Δ*H* < 0) and the unfavorable entropy loss (Δ*S* < 0) associated with the ordering of flexible, dynamic chains, which determines if the crystallization could occur or not and the magnitude of *T*_m_: Δ*G* = Δ*H − T*Δ*S*; at melting transition, Δ*G* = 0, *T* = *T*_m_ = Δ*H*_f_ / Δ*S*_f_. Although all methyl‐substituted nylon polymers in this series are atactic, α‐methyl substitution restricts local backbone rotations the most and thus experiences the smallest entropy penalty upon crystallization or the smallest entropy gain (positive Δ*S*_f_) upon melting, giving rise to the nylon with the highest *T*_m_ (200°C) and degree of crystallinity (*χ*_c_ = 21%) within this series. Moving to ε‐methyl substitution that also sufficiently restricts local backbone rotations, resulting in the nylon with the second highest *T*_m_ (166°C) and *χ*_c_ (17%). In comparison, β‐ and δ‐methyl groups only modestly perturb chain packing such that an entropy penalty is still small enough to permit crystallization, but their *T*_m_ (151, 145°C) and *χ*_c_ (16%, 12%) become lower. However, substituting the methyl group at the γ‐position places the methyl group the farthest away from both the carbonyl and amide functional groups (i.e., residing in the middle of the chain within the repeating unit), substantially increasing chain flexibility and conformational disorder; this increase, coupled with the configurational disorder of the side‐chain methyl groups for being atactic, imposes an entropy penalty that is too large to be compensated by the enthalpy gain due to hydrogen bonding and chain packing, thus yielding an amorphous polymer.

### 2.4 Chemical Recycling

**FIGURE 4:**
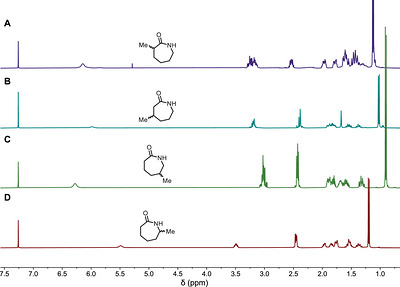
Demonstration of depolymerization of methyl‐substituted nylons to lactam monomers. ^1^H NMR spectra (CDCl_3_, 25°C) of monomers as recovered (i.e., unpurified, crude material) from the depolymerization: (A) recovered crude 7LM^αMe^; (B) recovered crude 7LM^βMe^; (C) recovered crude 7LM^δMe^; and (D) recovered crude 7LM^εMe^.

To establish chemical circularity for these new nylon‐6 variants, we investigated the chemical recyclability of nylon 6^αMe^, nylon 6^βMe^, nylon 6^δMe^, and nylon 6^εMe^ (Table S2). Each polymer can be efficiently depolymerized back to its corresponding monomer, 7LM^αMe^–7LM^εMe^, under vacuum (200 mTorr) at elevated temperatures (250°C–260°C) in the presence of 10 wt% *^t^* BuOK as an efficient catalyst, the conditions we and others had developed previously [35, 36]. As summarized in Table S1, the depolymerization afforded good to high isolated monomer yields, depending on the methyl‐substitution site: nylon 6^βMe^ gave the highest monomer recovery yield of 92%, followed by nylon 6^δMe^ (82%) and nylon 6^αMe^ (81%), while 6^εMe^ afforded the lowest yield of 77% in the series. The ^1^H NMR spectra of the recovered crude lactams show high purity and match those of the original monomers, confirming the success of the chemical depolymerization (Figure 4). Furthermore, the recovered monomer can be repolymerized to the corresponding nylons in 71%–89% yield with *M*_n_ from 11.5 to 34.3 kDa (Table S3). These results demonstrate that methyl‐substituted nylons can be chemically recycled in a closed‐loop process to regenerate their monomers.

## 3 Conclusions

This study reveals substantial and surprising effects of methyl substitution positions on the nylon‐6 backbone on crystallizability, thermal properties, mechanical performance, and recyclability of the resulting nylon‐6 variants. Four positional isomers, nylon 6^αMe^, nylon 6^βMe^, nylon 6^δMe^, and nylon 6^εMe^, were synthesized in good to high yields by AROP using simple, achiral bases, and all can be efficiently depolymerized back to their corresponding lactam monomers. Despite being atactic, these methyl‐substituted nylon‐6 variants can be effectively crystallized into crystalline domains, with thermal stability, melting temperatures, and mechanical properties strongly influenced by the position of the methyl substituent. Both crystallinity and melting temperature decrease from α‐ to β‐substitution to γ‐substitution (no crystallinity) and then recover at δ‐substitution toward ε‐substitution, highlighting the positional effect on chain packing and hydrogen‐bonding interactions.

Rationally designed control reactions confirm the absence of stereocontrol in the AROP of current 7LMs, establishing that crystallization in these monomethyl‐substituted nylon‐6 variants is not a result of the polymer stereoregularity but governed by the intimate interplay between the enthalpy gain due to enhanced hydrogen bonding and interchain interactions upon chain packing relative to the entropy penalty due to chain ordering. It should be clarified that “tacticity‐independent crystallinity” does not imply that tacticity has no influence on crystalline structure or thermal behavior. Rather, these results demonstrate that crystallization can occur even in the absence of stereoregularity, while increased stereoregularity remains associated with enhanced crystallinity and higher melting temperatures [19, 20, 21]. Overall, these findings not only expand the design for chemically recyclable nylons but also further illustrate a principle that crystalline order can persist even in atactic polymers when strong directional hydrogen bonds dominate the packing interactions.

## Author Contributions

**Lili Wang**: investigation, writing – review and editing. **Clarissa Lincoln**: investigation, writing – review and editing. **Nicholas A. Rorrer**: investigation. **Jun‐Jie Tian**: investigation, writing – original draft, writing – review and editing, conceptualization, methodology. **Ruirui Li**: investigation, writing – review and editing. **Jiyun Nam**: investigation, writing – review and editing. **Maëlle T. Gace**: investigation, writing –review and editing. **Andrea L. Baer**: investigation, writing – review and editing. **Eugene Y.‐X. Chen**: conceptualization, funding acquisition, writing – review and editing, project administration, supervision. **Wei‐Feng Zheng**: investigation, writing – review and editing.

## Conflicts of Interest

The authors declare no conflicts of interest.

## Supporting information




**Supporting File**: anie72417‐sup‐0001‐SuppMat.pdf.

## Data Availability

The data that support the findings of this study are available in the Supporting Information of this article.
