# Supplementary material for: A Circular and Tacticity‐Independent Crystalline Mono‐Substituted Nylon‐6 Platform: Unexpected Large Positional Effects on Crystallizability and Performance
Source: Angew Chem Int Ed Engl. 2026 May 2;65(25):e9256032. doi: 10.1002/anie.9256032 (PMC13266927; doi:10.1002/anie.9256032)
Supplement: Supplementary file 1 — Supporting File: anie72417‐sup‐0001‐SuppMat.pdf. [file ANIE-65-e9256032-s001.pdf]

Supporting Information

A Circular and Tacticity-Independent Crystalline Mono-Substituted Nylon-6 Platform: Unexpected Large Positional Effects on Crystallizability and Performance

Jun-Jie Tian, Jiyun Nam, Lili Wang, Andrea L. Baer, Ruirui Li, Maëlle T. Gace, Wei-Feng Zheng, Clarissa Lincoln, Nicholas A. Rorrer, Eugene Y.-X. Chen

Table of Contents

Materials and General Methods **2**

General Polymerization Procedures **2**

Chemical Recycling to Monomer **2**

Molar Mass Measurements **3**

Spectroscopic Characterizations **3**

Thermal Analysis **4**

Tensile and Dynamic Mechanical Analysis **4**

Monomer Synthesis **5**

Control Reactions **7**

Supplementary Figures **8**

Figure S1–S8. ^{1}H and ^{13}C NMR spectra of small molecules. 8

Figure S9–S12. ^{1}H and ^{13}C NMR spectra of methyl nylon-6 variants. 16

Figure S13–S24. SEC chromatogram 20

Figure S25–S34. TGA/DTG and DSC 26

Figure S35–S38. Wide-angle X-ray scattering (WAXS) profiles 31

Figure S39. Small-angle X-ray scattering (SAXS). 33

Figure S40–S43. Tensile curves. 34

Figure S44–S47. Overlay plots of storage modulus E', loss modulus E'', and tan δ (E''/E') 36

Figure S48. Water absorption after 7 days of immersion. 38

Supplementary Tables **39**

Table S1. Polymerization of (R)-7LM$^{\beta Me}$ and 7LM$^{\beta Me}$. 39

Table S2. Results of the depolymerization of methyl nylon-6 variants. 39

Table S3. Polymerization of recovered 7LM^{Me}. 39

Table S4–S7. Tensile stress-strain data of nylon dog-bone-shaped specimens 40

References **41**

Materials and General Methods

All syntheses and manipulations of air- and moisture-sensitive materials were carried out in flamed Schlenk-type glassware on a dual-manifold Schlenk line, on a high-vacuum line, or in an N_2-filled glovebox. High-performance liquid chromatography (HPLC)-grade organic solvents were first sparged extensively with nitrogen during filling of 20 L solvent reservoirs and then dried by passage through activated alumina. For the *N,N*-dimethylacetamide (DMAc) used in polymerization reactions, HPLC-grade DMAc was degassed and dried over activated Davison 4 Å molecular sieves for 3 days.

Sodium hydride (NaH, 60 wt% dispersion in mineral oil) and potassium *tert*-butoxide (^{t}BuOK) were purchased from TCI Chemical Co., while superbase ^{t}Bu-P$_4$ {[(Me$_2$N)$_3$P=N)]$_3$P=N(^{t}Bu)} (0.8 M in hexane) was purchased from Sigma-Aldrich Chemical Co.; all the above reagents were used as received. All other chemicals were purchased from their respective commercial sources: sodium hydroxide, 2-methylcyclohexanone, and 3-methylcyclohexanone were purchased from Fisher Scientific Co.; 1,1,1,3,3,3-hexafluoroisopropanol (HFIP), hydroxylamine-*O*-sulfonic acid, and formic acid were purchased from Oakwood Chemical Co. and used as received.

General Polymerization Procedures

Inside the glovebox, a 10 mL Schlenk flask was loaded with a methyl ε-caprolactam (7LM^{Me}), the corresponding activator, and solvent DMAc (if used). The mixture was brought to the desired temperature before the addition of the base catalyst. To initiate polymerization, ^{t}Bu-P4 (0.8 M in hexane) or NaH (60 wt% dispersion in mineral oil) was added to the stirred mixture, and the flask was capped. After a desired period, the polymerization was quenched by adding 0.5 mL of formic acid, then dissolved in HFIP, followed by precipitation in diethyl ether 2-3 times. After filtration, the polymer was dried in vacuo at 100 °C to a constant weight.

Chemical Recycling to Monomer

Inside the glovebox, 254 mg nylon 6^{Me} was added to a glass sublimator with a cooling water condenser. The powder or fine granules were pre-wet with anhydrous tetrahydrofuran (THF, 0.5 mL) and then coated in a ^{t}BuOK-THF solution (25.4 mg ^{t}BuOK in 1.0 mL THF). The sublimator was sealed and attached to the Schlenk line, where the vacuum was pulled carefully to evaporate the THF. It was necessary to open the apparatus briefly to move all the polymer to the bottom and

clean the condenser. After re-assembly, the condenser lines were opened, and the temperature of the thermocouple-controlled heating mantle was held at a given temperature under vacuum (200 mTorr). The crude product was rinsed into a round-bottom flask with dichloromethane (DCM), which was removed by rotary evaporation and weighed for monomer yield.

Molar Mass Measurements

Weight-average molar mass (M_w), number-average molar mass (M_n), and dispersity ($Đ$) values were determined by size exclusion chromatography (SEC) on an Agilent 1260 Infinity II LC system coupled with a Wyatt Technology miniDAWN TREOS Multi-Angle Light scattering detector and a Wyatt Technology Optilab T-rEX differential refractometer. Note: samples in Table S3 were measured with Wyatt Technology miniDAWN Multi-Angle Light scattering detector and a Wyatt Technology Optilab differential refractometer detector. The SEC analysis was performed at 40 °C with a flow rate of 0.35 mL/min using three Agilent PL HFIP gel 250 × 4.6 mm columns and a matching guard column attached in series. The mobile phase consisted of 0.1 µm filtered HPLC-grade HFIP amended with 20 mM sodium trifluoroacetate. Wyatt Technology Astra 8.2.0 molecular weight characterization software was used for data analysis. The dn/dc was calculated assuming 100% mass recovery, and low molar mass peak tails beyond the column resolution limit were not included in peak integration. The chromatograms are plotted with signals from the differential refractometer (Figures S13–S24). Nylon $6^{\varepsilon Me}$ (103 kDa, $Đ$ = 2.55) sample was prepared at ~2.5 mg/mL, and the other samples were prepared at ~5 mg/mL.

Spectroscopic Characterizations

NMR spectra were recorded on a Varian Inova or Bruker AV-III 400 MHz spectrometer (400 MHz, ^{1}H; 101 MHz, ^{13}C) at 298 K. Chemical shifts (δ) are reported in ppm with the solvent resonance employed as the internal standard (chloroform-d_1 at 7.26 ppm for ^{1}H NMR and 77.2 ppm for ^{13}C NMR; dimethyl sulfoxide (DMSO)-d_6 at 3.33 and 2.50 ppm for ^{1}H NMR and 39.5 ppm for ^{13}C NMR; Trifluoroacetic acid (TFA)-d_1) at 11.50 ppm for ^{1}H NMR and 164.2, 116.6 ppm for ^{13}C NMR). Signals are reported as integration, multiplicity (s = singlet, d = doublet, t = triplet, q = quartet, m = multiplet, br = broad signal), coupling constant (J) in Hz, and assignment.

X-ray scattering. Wide-angle X-ray scattering (WAXS) and small-angle X-ray scattering (SAXS) were performed with Xenocs Xeuss 3.0 SAXS/WAXS. The X-Ray beam energy was 8048 keV (Cu Kα, 1.5418 Å) with a beam size of 0.7 (horizontal) × 0.7 (vertical) mm^2 for the slits closest to

the sample. The images were taken with an Eiger2 R 1M (Dectris) area detector comprising (1028 × 1062) pixels with a pixel size of 75 μm^2 in transmission geometry. The sample-to-detector distance was 1100 mm (SAXS), and silver behenate (AgBe) was used as the standard to calibrate the sample-to-detector distance. The 2D scattering patterns were azimuthally integrated to afford one-dimensional profiles presented as scattering vector (q) versus scattering intensity, where the magnitude of the scattering vector is calculated with $q = (4\pi/\lambda) \sin(\theta/2)$. For WAXS analysis, the sample-to-detector distance was 42.5 mm, and lanthanum hexaboride (LaB_6) was used as the standard to calibrate the sample-to-detector distance. Degree of crystallinity (χ_c) was calculated as $(A_{total}-A_{amorphous})/A_{total}$, which indicated the ratio of the area of the crystalline fraction ($A_{crystalline} = A_{total}-A_{amorphous}$) to the total area ($A_{total} = A_{amorphous} + A_{crystalline}$) based on peak-area deconvolution of the WAXS profile.

Thermal Analysis

Melting-transition temperature (T_m) and glass-transition (T_g) temperature were measured by differential scanning calorimetry (DSC) on an Auto Q20, TA Instrument. Both heating rate and cooling rate were 10 °C/min unless indicated otherwise. Decomposition temperatures (T_d, defined by the temperature of 5% weight loss) and maximum rate decomposition temperatures (T_{max}) of the polymers were measured by thermalgravimetric analysis (TGA) on a Q50 TGA Analyzer, TA Instrument. Polymer samples were heated from ambient temperature to 700 °C at a heating rate of 10 °C/min. Values of T_{max} were obtained from derivative (wt %/°C) *vs.* temperature (°C) plots.

Tensile and Dynamic Mechanical Analysis

Tensile stress/strain testing was performed by an Instron 5966 universal testing system (10 kN load cell) on dog-bone-shaped test specimens (ASTM D638 standard; Type V) prepared *via* compression molding using a Carver Bench Top Laboratory Press (Model 4386) equipped with a two-column hydraulic unit (Carver, Model 3912, maximum force 24000 psi) unless indicated otherwise. Isolated polymer materials were loaded between non-stick Teflon paper sheets into a stainless-steel mold with inset dimensions 30 × 73.5 × 0.38 mm fabricated in-house and compressed between two 6" × 6" steel electrically heated platens (EHP) clamp force 3000 psi, at 210 °C for nylon $6^{\alpha Me}$, 180 °C for nylon $6^{\beta Me}$, nylon $6^{\delta Me}$, and nylon $6^{\varepsilon Me}$ specimens, followed by slow cooling to yield the compression molding film. Specimens for analysis were generated *via* compression molding and cut using an ASTM D638-5-IMP cutting die (Qualitest) to standard

dimensions. Mechanical behavior was averaged for all the specimens measured for each species investigated. Thickness (0.35–0.40 mm), width (3.18 mm), and grip length (26.4 ± 0.2 mm) of the measured dog-bone specimens were measured for normalization of data by the Bluehill measurement software (Instron). Test specimens were affixed to the screw-tight grip frame. Tensile stress and strain were measured to the point of material break at a grip extension speed of 5.0 mm min^{-1} at ambient conditions. Testing of control standards of commercial nylon 6 (M_n = 14.0 kDa, M_w = 21.5 kDa, $Đ$ = 1.54, purchased from Sigma-Aldrich) for comparative stress-strain curves is included in Figure 2E; the detailed tensile testing results (individual stress-strain curves and tables) were previously reported,[1] and the values were taken from that paper for comparison while plotting overlay Figure 2E in the main text.

Storage modulus (E'), loss modulus (E''), and tan δ (E''/E') were measured by dynamic mechanical analyzer (DMA) on a Q800 DMA Analyzer (TA Instruments) in a tension film mode at a maximum strain of 0.1% and a frequency of 1 Hz (complying with strain-sweep and frequency-sweep linearity analysis performed before sample testing). Specimens for analysis were generated via compression molding and cut down to a standard width (5.4 mm). Specimen length (13 mm) and thickness (0.38–0.40 mm) were measured for normalization of data by Q-series measurement software (TA Instruments). Test specimens were mounted to screw-tight grips (maximum 2 N). The samples were heated from -20 °C to 150 °C at a heating rate of 3 °C min^{-1}. The α-transition temperature was calculated as the peak maxima of the tan δ curve.

Monomer Synthesis

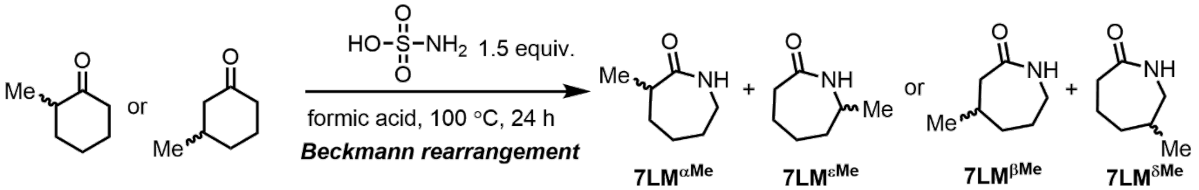

Preparation of $7LM^{Me}$ ($7LM^{\alpha Me}$, $7LM^{\beta Me}$, $7LM^{\delta Me}$, and $7LM^{\varepsilon Me}$) via Beckmann rearrangement. A solution of 2- or 3-methylcyclohexanone (22.4 g, 200 mmol, 1.0 equiv) in formic acid (100 mL) was added dropwise to a solution of hydroxylamine-O-sulfonic acid (33.9 g, 300 mmol, 1.5 equiv) in formic acid (200 mL) and stirred at 23 °C for 30 min. The mixture was then heated to 100 °C for 24 h. After cooling to room temperature, the reaction was concentrated under reduced pressure and quenched with 5 N NaOH (400 mL). The aqueous phase was extracted with ethyl acetate (3 × 500 mL), and the combined organic layers were washed with brine (200 mL), dried over Na_2SO_4,

filtered, and concentrated under vacuum to afford a crude residue. Purification was achieved by column chromatography or recrystallization from dichloromethane/hexane, followed by vacuum sublimation to give pure $7LM^{Me}$. From 2-methylcyclohexanone, a mixture of $7LM^{\alpha Me}$ and $7LM^{\varepsilon Me}$ was obtained in 84% combined yield (α:ε = 1:9); $7LM^{\alpha Me}$ was isolated by column chromatography, which can also be synthesized by α-methylation of 7LM according to the previous literature;[1] $7LM^{\varepsilon Me}$ was obtained by recrystallization from DCM/hexanes. From 3-methylcyclohexanone, a mixture of $7LM^{\beta Me}$ and $7LM^{\delta Me}$ was obtained in 95% combined yield (β:δ = 1.1:1); $7LM^{\beta Me}$ was isolated by recrystallization from DCM/hexanes, and $7LM^{\delta Me}$ was isolated by column chromatography.

(*R*)-$7LM^{\beta Me}$ was synthesized according to the previous literature.[2] Activators (**A1**, *rac*-**A2,** and (*R*)-**A2**) were synthesized according to the previous literature.[1]

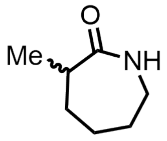

3-Methylazepan-2-one ($7LM^{\alpha Me}$). White solid. ^{1}H NMR (400 MHz, $CDCl_3$) δ 6.13 (br, 1H), 3.31 – 3.12 (m, 2H), 2.59 – 2.48 (m, 1H), 2.04 – 1.92 (m, 1H), 1.84 – 1.73 (m, 1H), 1.66 – 1.56 (m, 2H), 1.50 – 1.36 (m, 2H), 1.13 (d, *J* = 6.8 Hz, 3H). ^{13}C NMR (101 MHz, $CDCl_3$) δ 180.56, 42.38, 38.28, 32.34, 30.00, 29.64, 17.75.

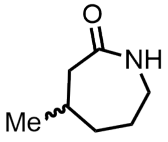

4-Methylazepan-2-one ($7LM^{\beta Me}$). White solid. ^{1}H NMR (400 MHz, $CDCl_3$) δ 6.51 (br, 1H), 3.26 – 3.09 (m, 2H), 2.46 – 2.26 (m, 2H), 1.94 – 1.71 (m, 3H), 1.61 – 1.47 (m, 1H), 1.41 – 1.27 (m, 1H), 1.01 (d, *J* = 6.8 Hz, 3H). ^{13}C NMR (101 MHz, $CDCl_3$) δ 177.90, 44.27, 42.87, 39.05, 29.29, 28.69, 23.04.

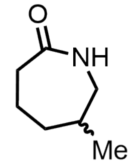

6-Methylazepan-2-one ($7LM^{\delta Me}$). White solid. ^{1}H NMR (400 MHz, $CDCl_3$) δ 6.27 (br, 1H), 3.04 – 2.89 (m, 2H), 2.46 – 2.29 (m, 2H), 1.90 – 1.72 (m, 2H), 1.69 – 1.48 (m, 2H), 1.33 – 1.19 (m, 1H), 0.85 (d, *J* = 6.9 Hz, 3H). ^{13}C NMR (101 MHz, $CDCl_3$) δ 179.03, 49.09, 38.80, 36.65, 34.37, 22.18, 19.48.

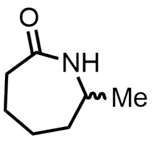

7-Methylazepan-2-one ($7LM^{\varepsilon Me}$). White solid. ^{1}H NMR (400 MHz, $CDCl_3$) δ 5.97 (br, 1H), 3.52 – 3.39 (m, 1H), 2.45 – 2.39 (m, 2H), 1.97 – 1.89 (m, 1H), 1.84 – 1.77 (m, 1H), 1.76 – 1.69 (m, 1H), 1.56 – 1.46 (m, 2H), 1.40 – 1.28 (m, 1H), 1.18 (d, *J* = 6.7 Hz, 3H). ^{13}C NMR (101 MHz, $CDCl_3$) δ 177.90, 49.49, 37.66, 37.10, 29.86, 23.17, 22.58.

Control Reactions

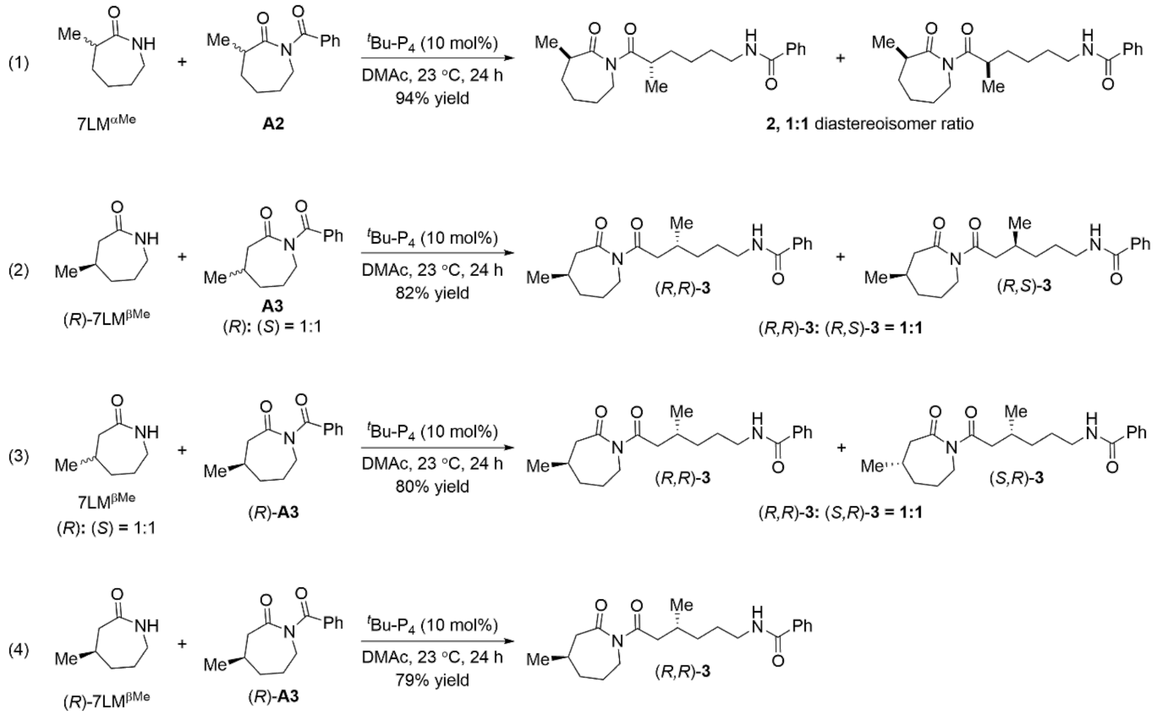

Inside the glovebox, a 10 mL Schlenk flask was loaded with 7LM$^{\alpha Me}$ or 7LM$^{\beta Me}$ (25.4 mg, 0.2 mmol, 1.0 equiv), the corresponding activator (46.2 mg, 0.2 mmol, 1.0 equiv), and DMAc (1 mL). ^{t}Bu-P_4 (0.8 M in hexane, 0.02 mmol, 10 mol%) was then injected into the stirring mixture, and the flask was capped. After 24 h at 23 °C, the polymerization was quenched by the addition of benzoic acid in $CHCl_3$ (10 mg/mL). The residue was purified by column chromatography using EA/hexane as eluent for further characterization.

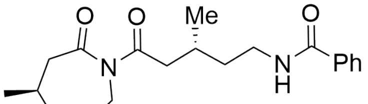

***N*-((*R*)-3-methyl-5-((*R*)-4-methyl-2-oxoazepan-1-yl)-5-oxopentyl)benzamide [(*R*,*R*)-3].** Colorless oil. ^{1}H NMR (400 MHz, $CDCl_3$) δ 7.83 – 7.75 (m, 2H), 7.47 (dd, *J* = 8.7, 5.9 Hz, 1H), 7.45 – 7.37 (m, 2H), 6.53 (s, 1H), 4.36 (dd, *J* = 14.9, 7.0 Hz, 1H), 3.62 – 3.50 (m, 1H), 3.41 – 3.31 (m, 2H), 2.90 (dd, *J* = 16.4, 6.8 Hz, 1H), 2.74 – 2.61 (m, 2H), 2.60 – 2.52 (m, 1H), 2.13 – 2.03 (m, 1H), 1.94 – 1.80 (m, 3H), 1.73 – 1.54 (m, 2H), 1.50 – 1.19 (m, 4H), 1.02 (d, *J* = 6.7 Hz, 3H), 0.94 (d, *J* = 6.7 Hz, 3H). ^{13}C NMR (101 MHz, $CDCl_3$) δ 176.67, 175.82, 167.53, 134.97, 131.34, 128.55, 127.08, 47.39, 46.52, 43.14, 39.67, 37.59, 33.77, 30.21, 29.18, 27.51, 26.71, 22.91, 19.86.

Supplementary Figures

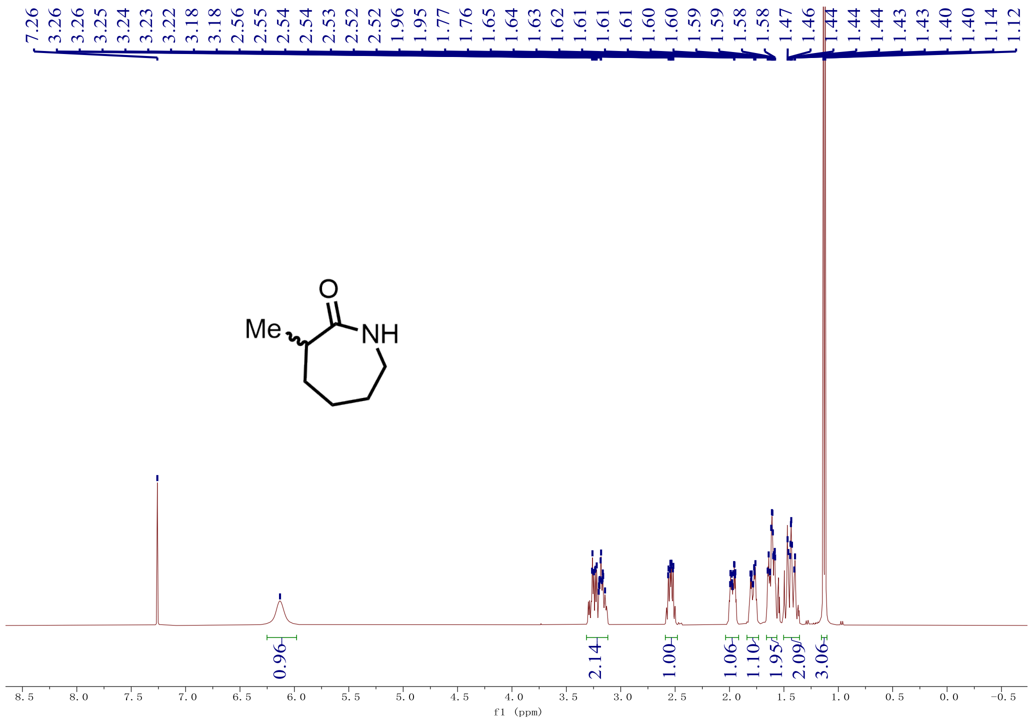

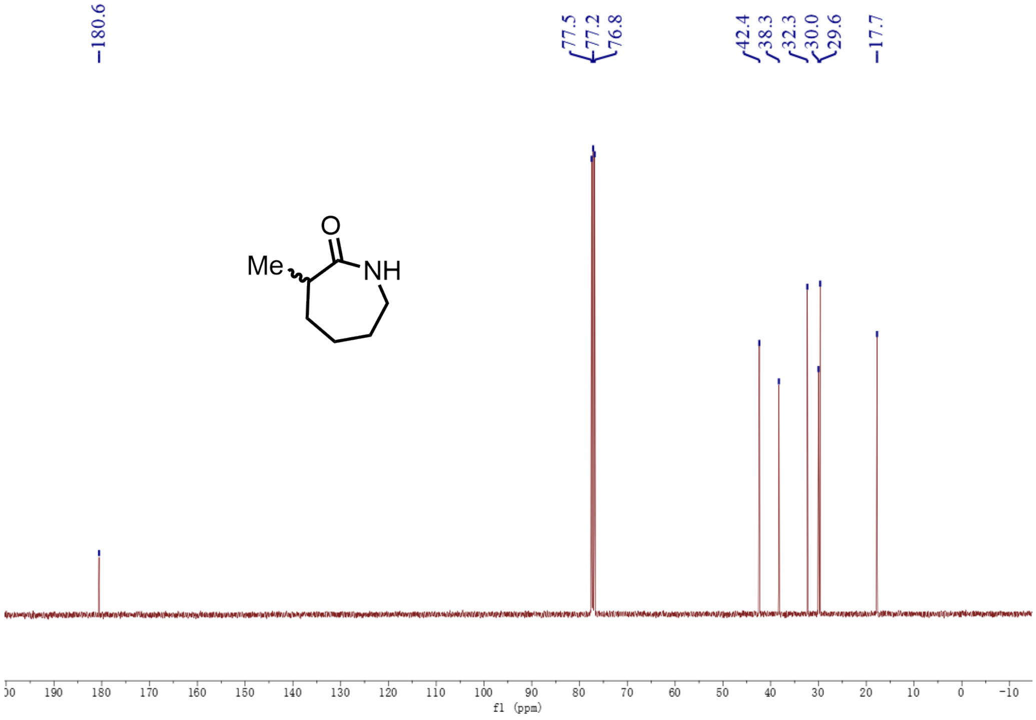

Figure S1. ^{1}H and ^{13}C NMR ($CDCl_3$, 25 °C) spectra of $7LM^{\alpha Me}$.

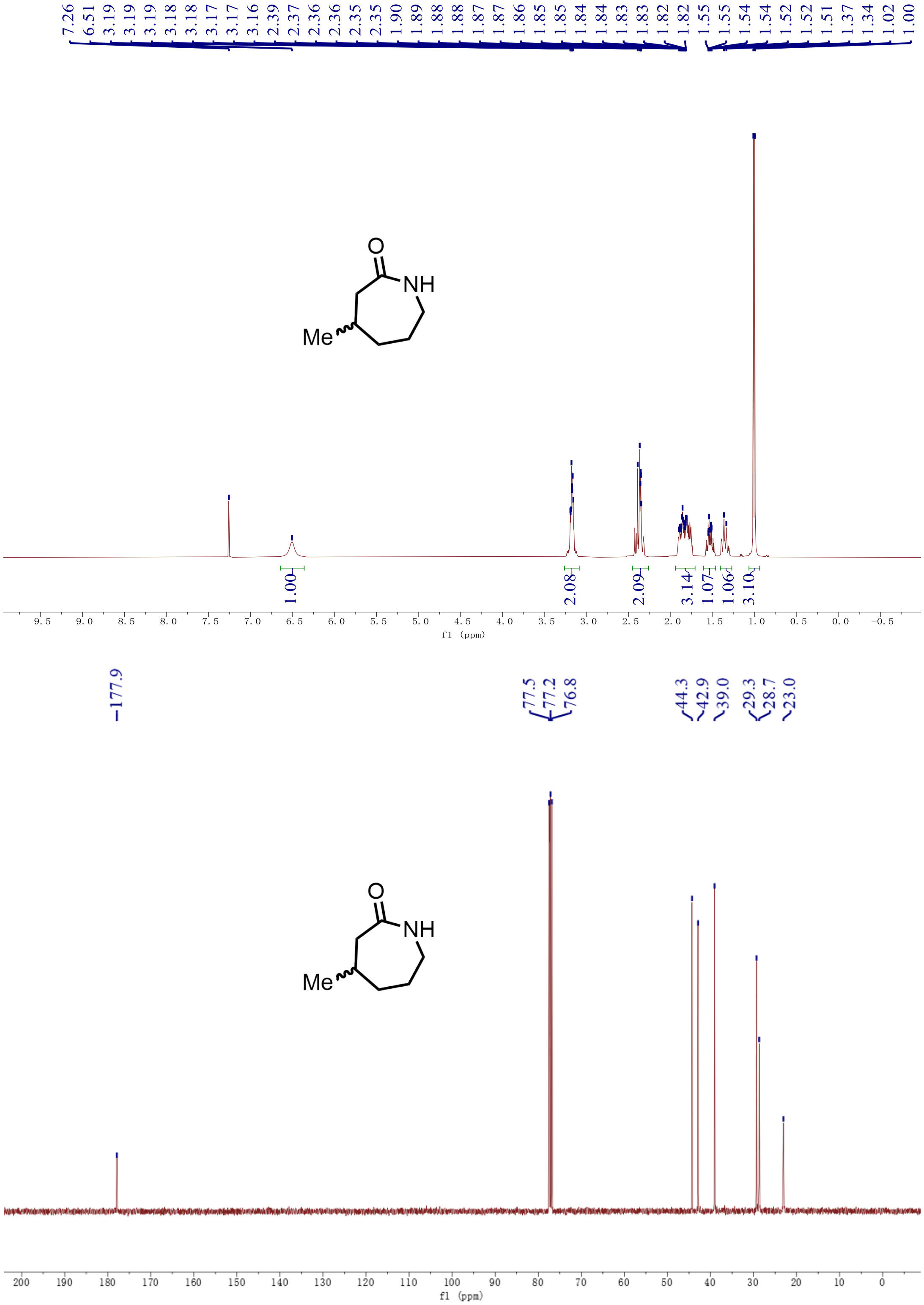

Figure S2. ^{1}H and ^{13}C NMR ($CDCl_3$, 25 °C) spectra of $7LM^{\beta Me}$.

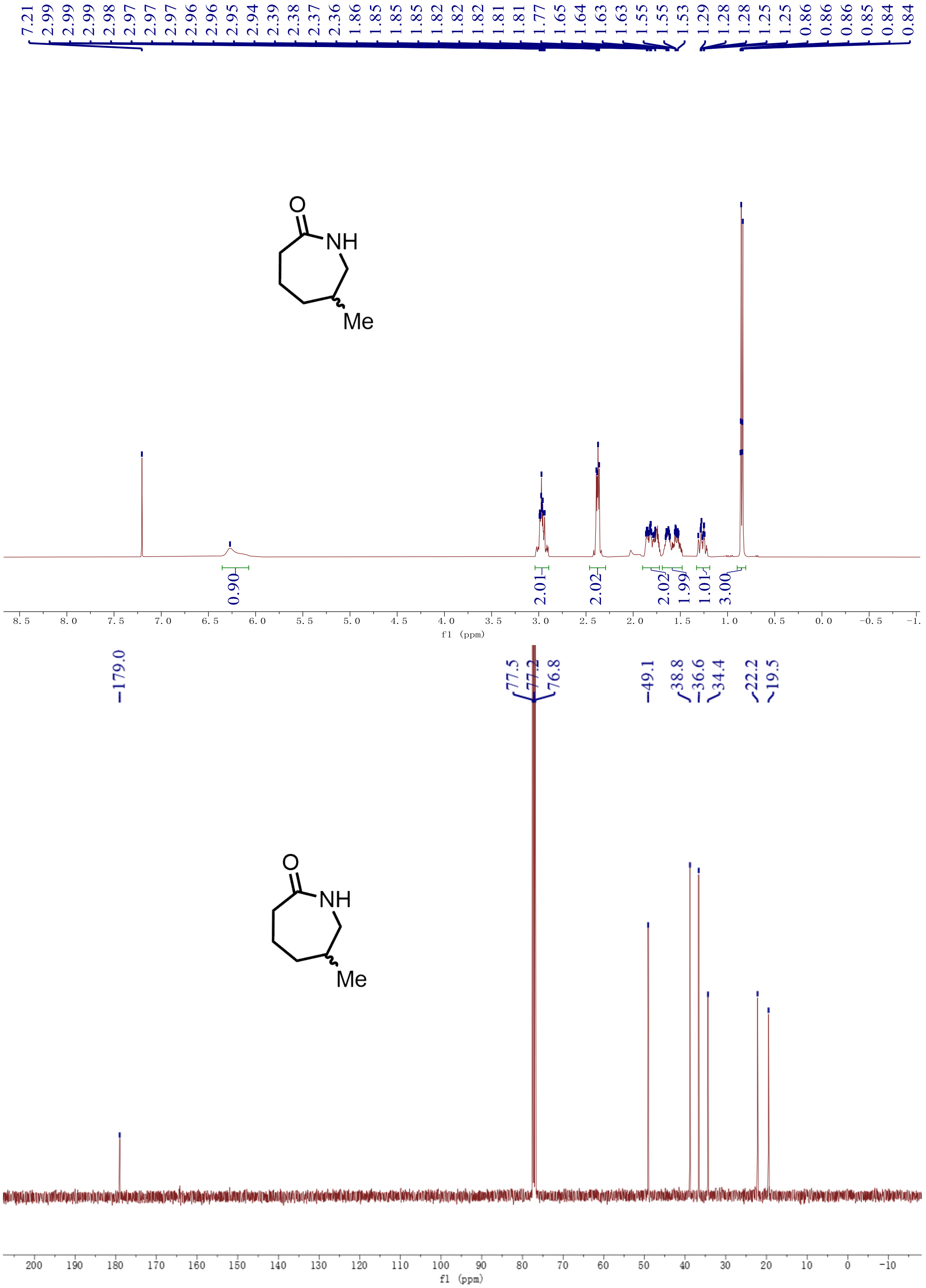

Figure S3. ^{1}H and ^{13}C NMR ($CDCl_3$, 25 °C) spectra of $7LM^{\delta Me}$.

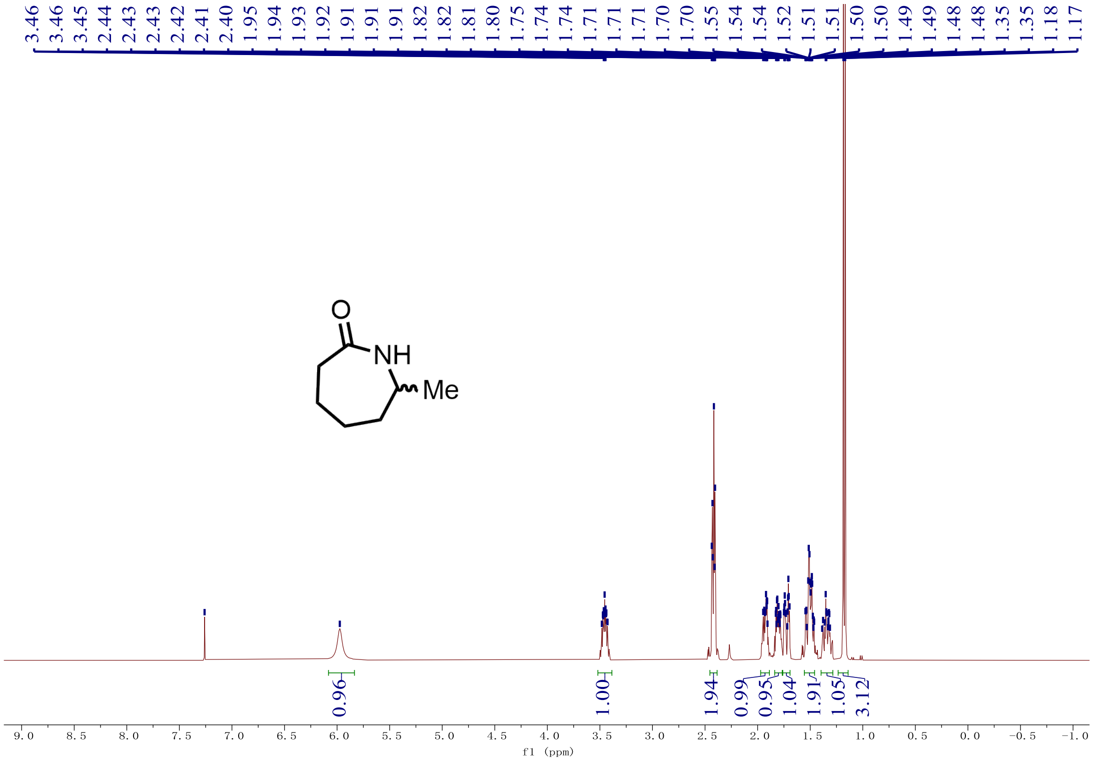

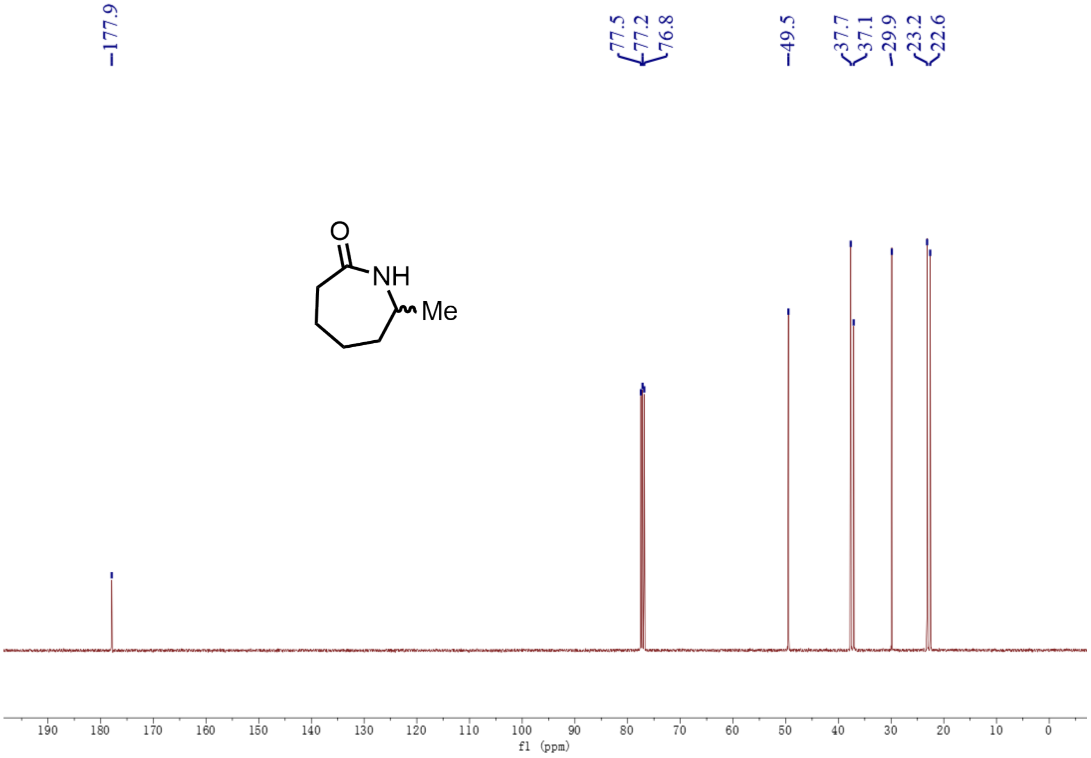

Figure S4. ^{1}H and ^{13}C NMR ($CDCl_3$, 25 °C) spectra of 7LM$^{\varepsilon Me}$.

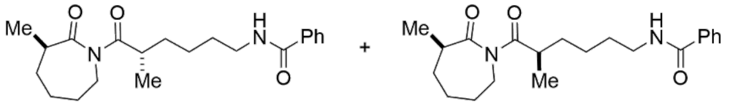

2, 1:1 diastereoisomer ratio

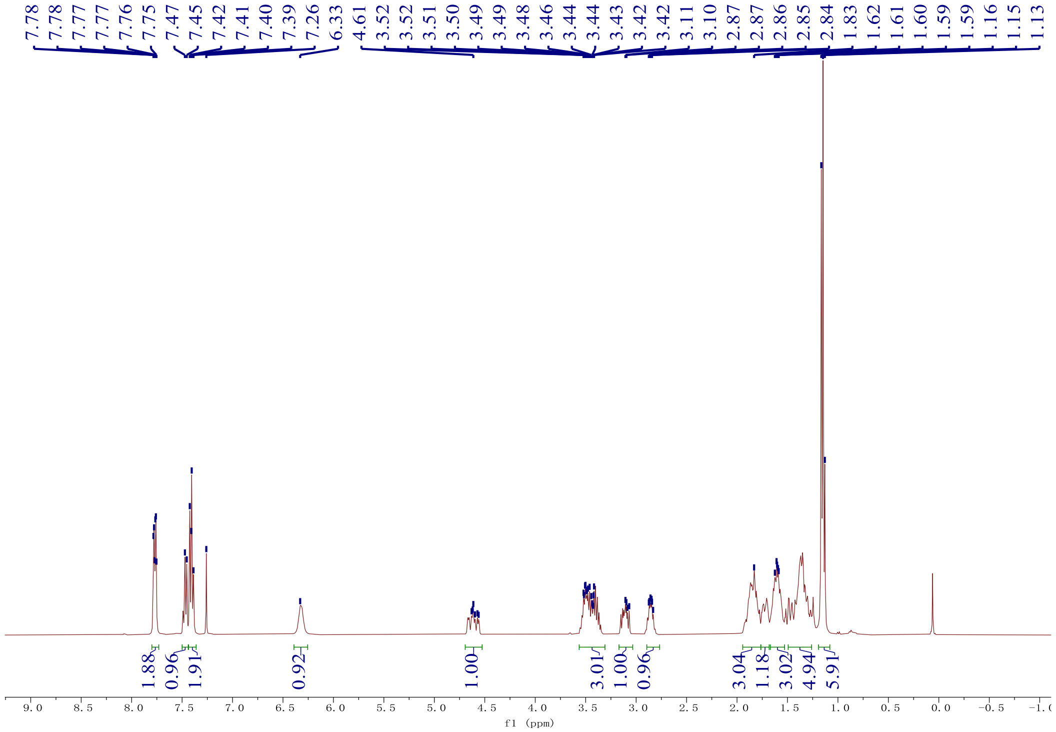

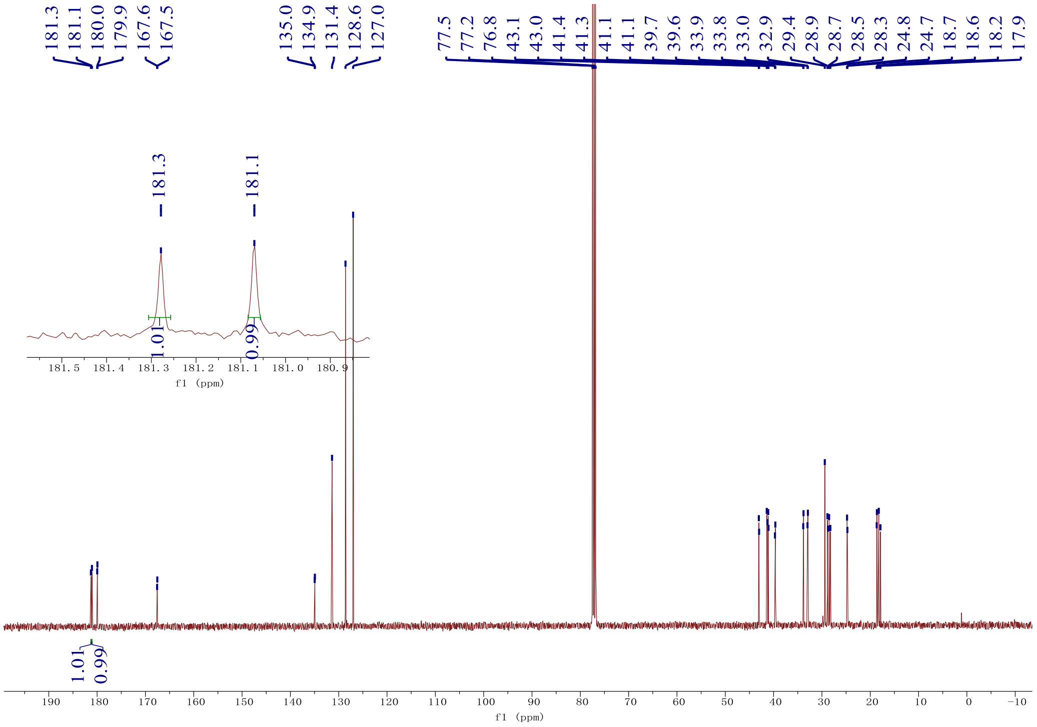

Figure S5. ^{1}H and ^{13}C NMR ($CDCl_3$, 25 °C) spectra of **2**.

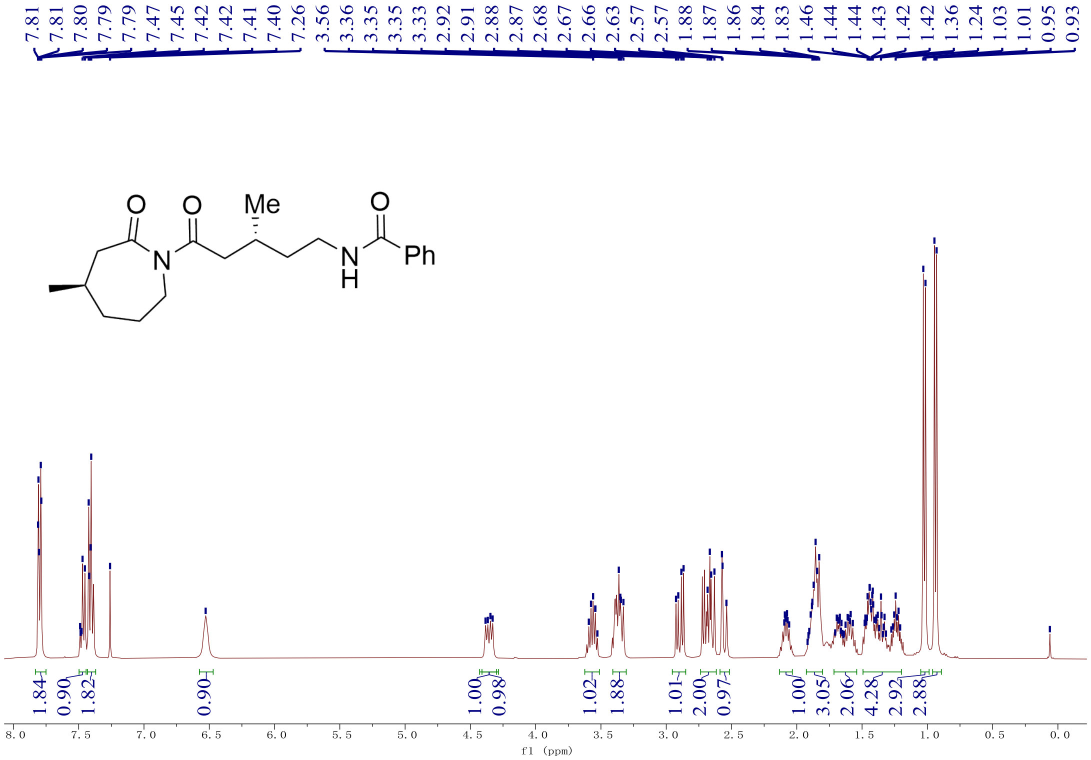

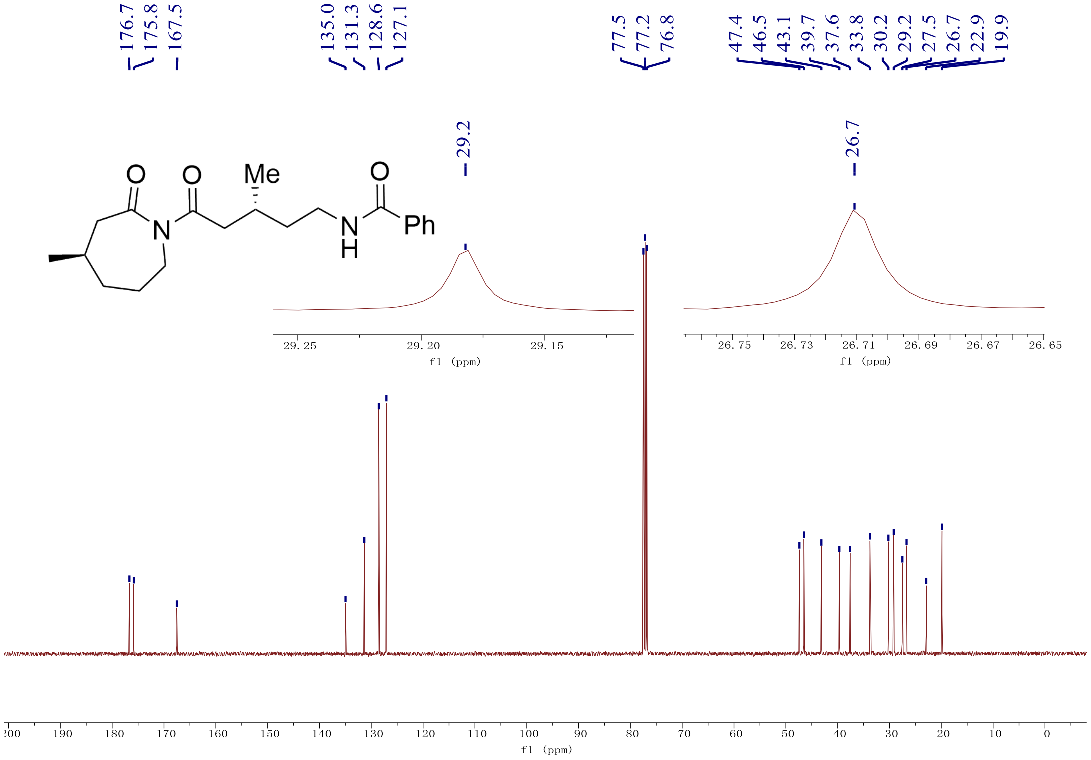

Figure S6. ^{1}H and ^{13}C NMR ($CDCl_3$, 25 °C) spectra of (*R*,*R*)-**3**.

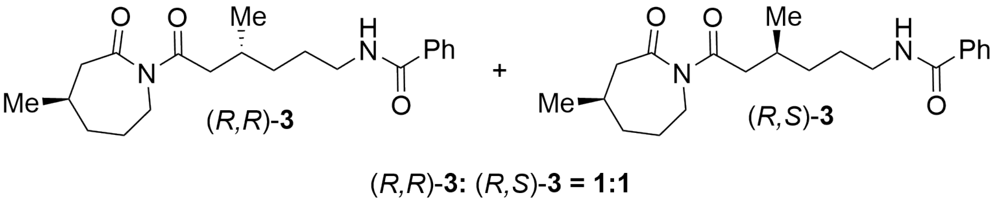

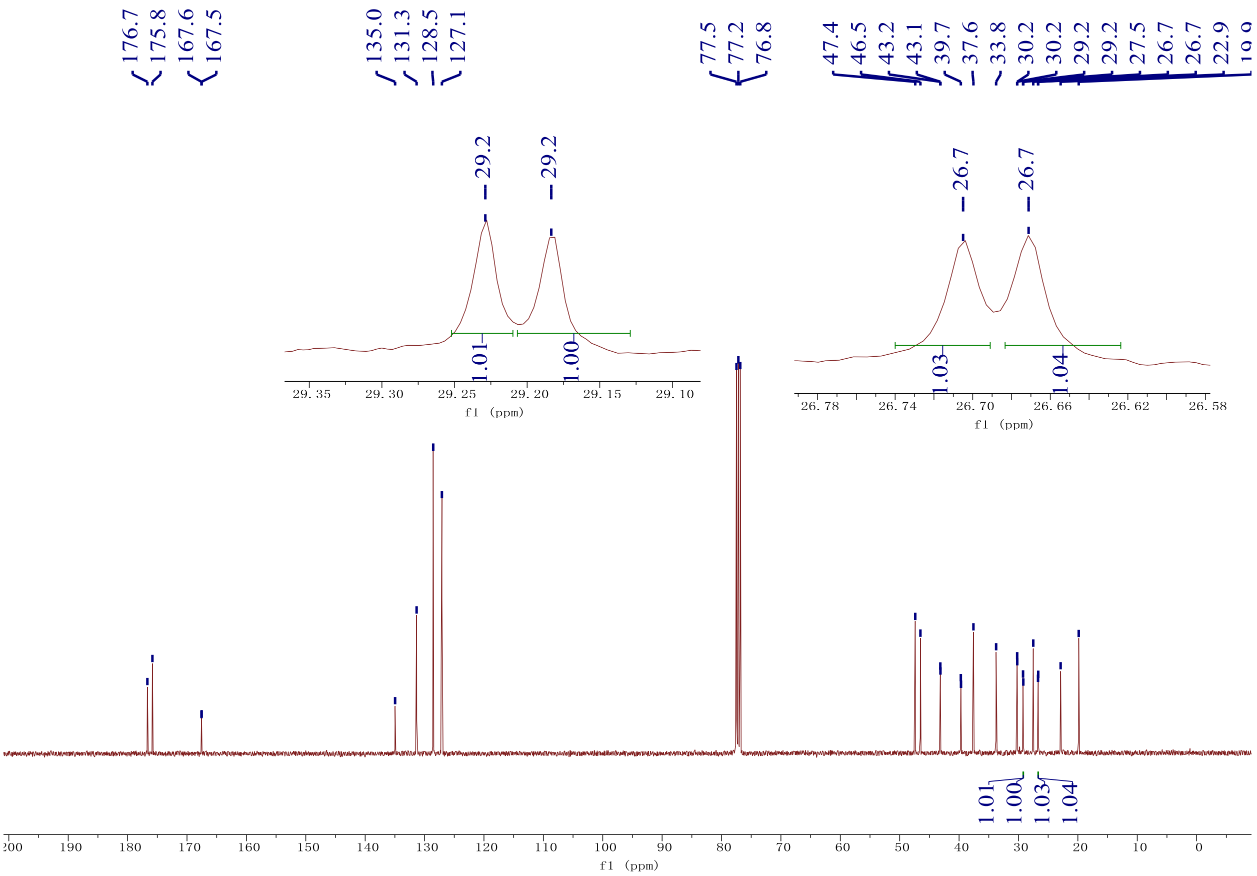

Figure S7. ^{1}H NMR ($CDCl_3$, 25 °C) spectra of a mixture of (*R*,*R*)-**3**: (*R*,*S*)-**3**.

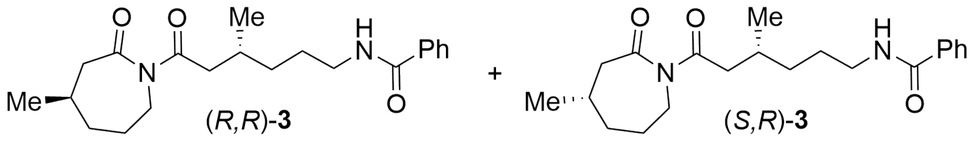

(*R*,*R*)-**3**: (*S*,*R*)-**3** = **1:1**

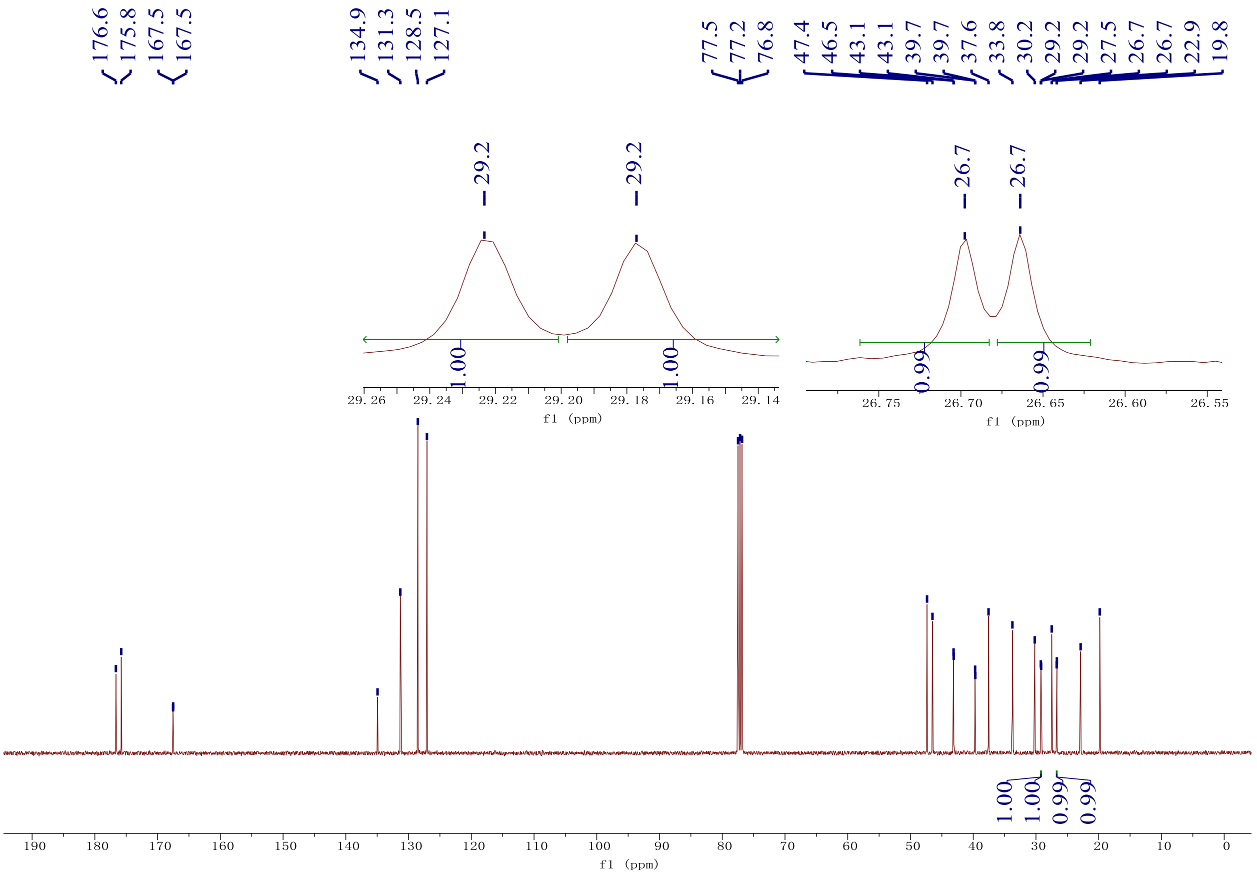

Figure S8. ^{1}H NMR ($CDCl_3$, 25 °C) spectra of a mixture of (*R*,*R*)-**3**: (*S*,*R*)-**3**.

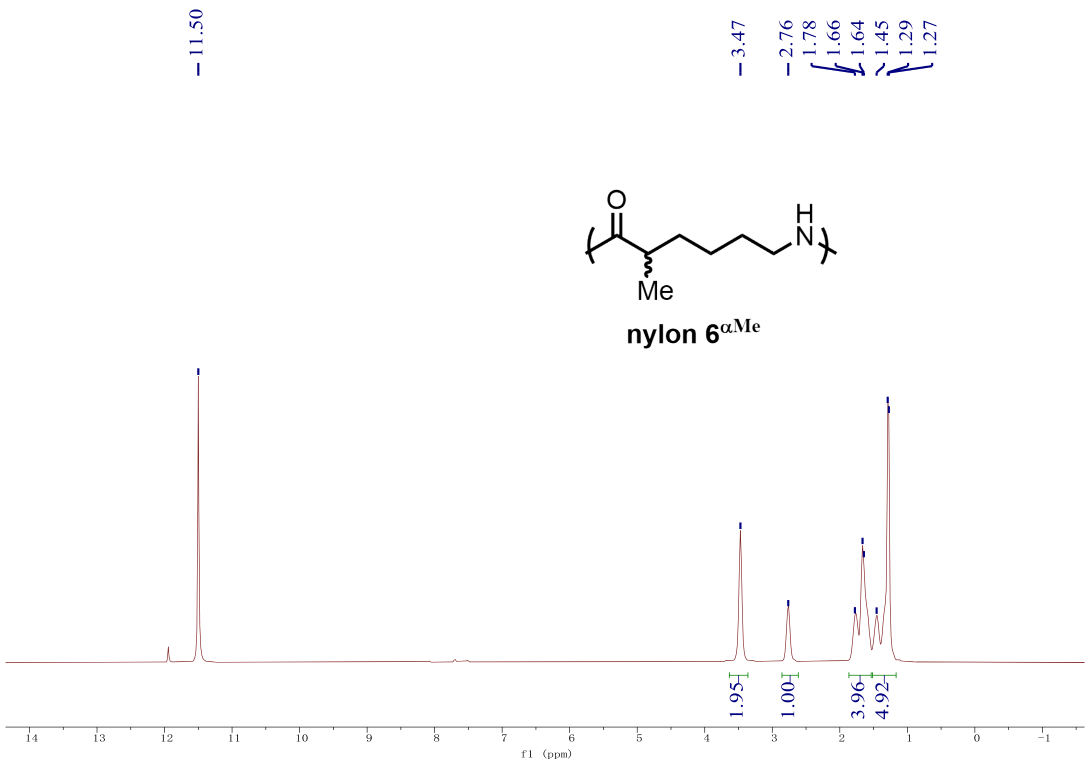

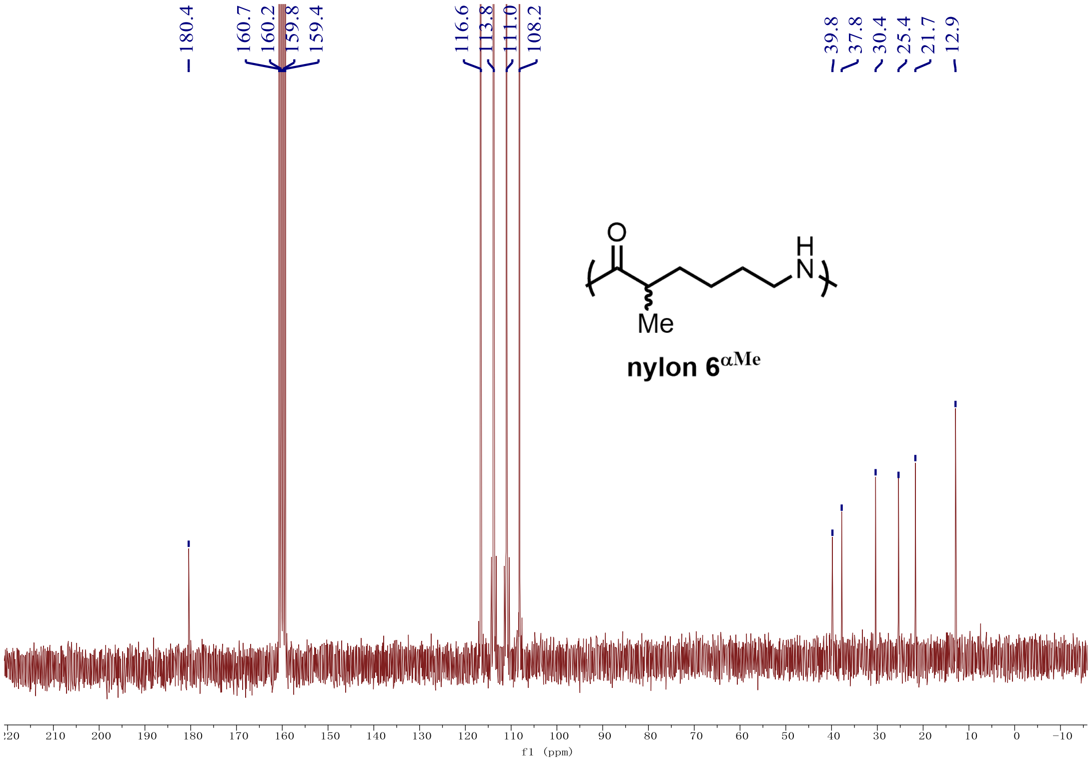

Figure S9. ^{1}H and ^{13}C NMR (TFA-d_1, 25 °C) spectra of nylon $6^{\alpha Me}$.

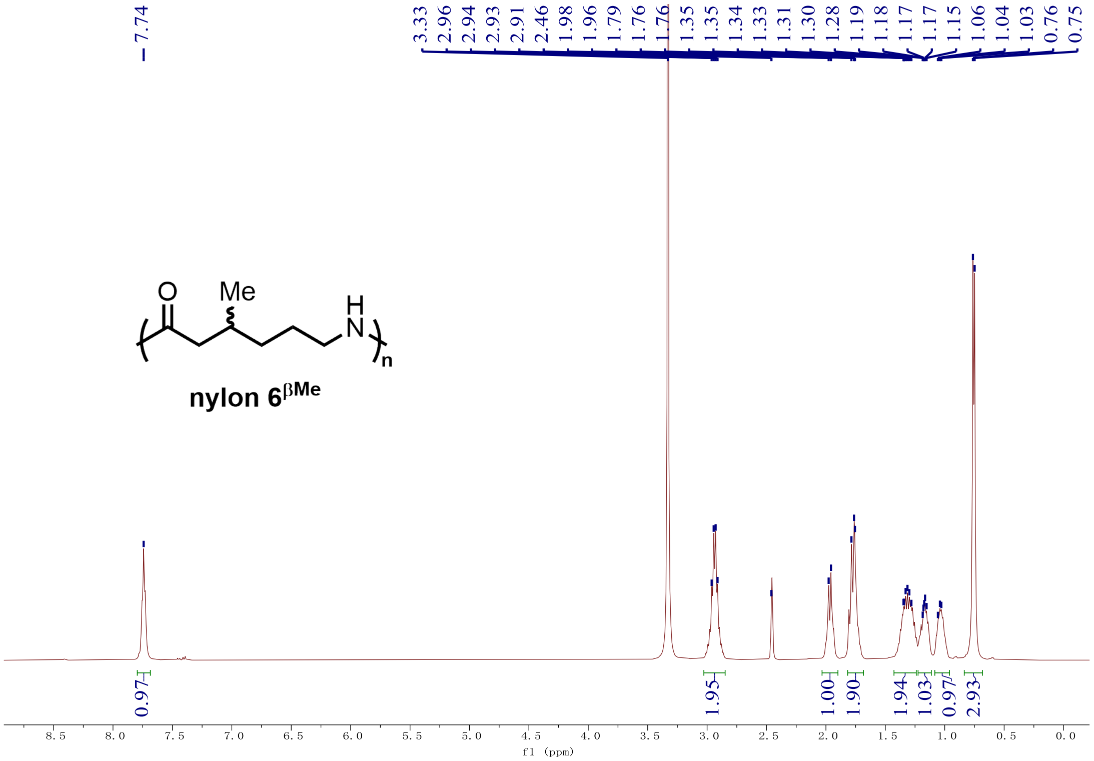

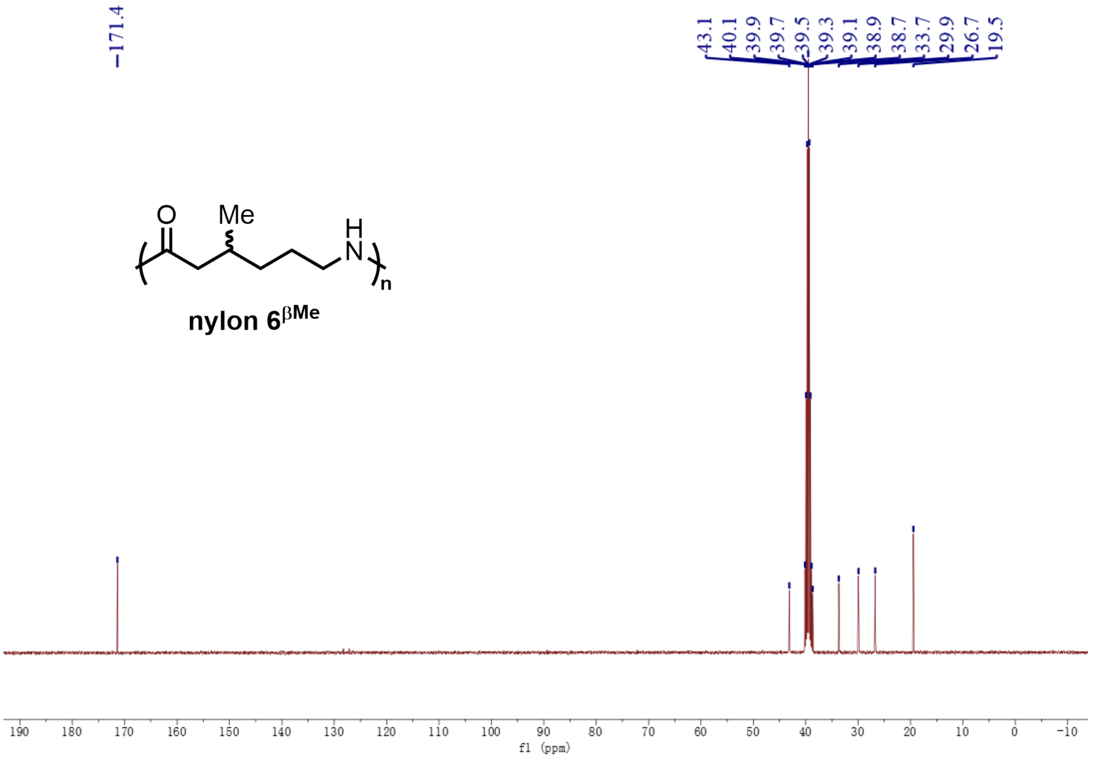

Figure S10. ^{1}H and ^{13}C NMR (DMSO-d_6, 25 °C) spectra of nylon $6^{\beta Me}$.

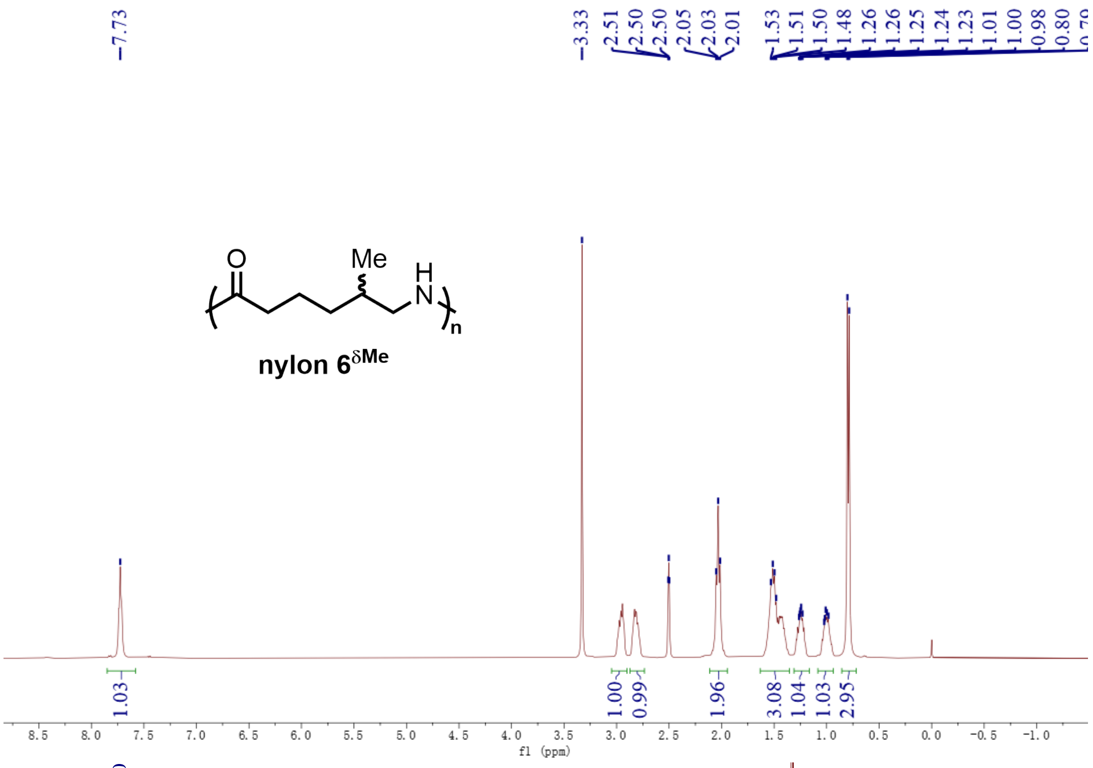

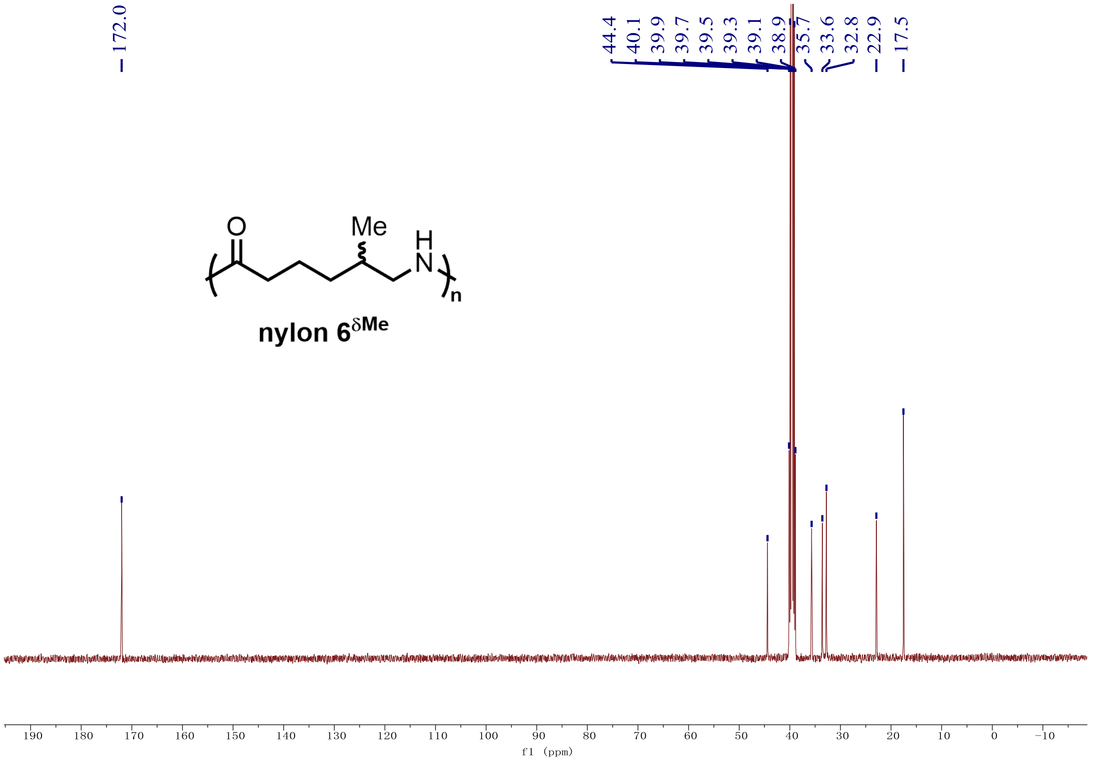

Figure S11. ^{1}H and ^{13}C NMR (DMSO-d_6, 25 °C) spectra of nylon $6^{\delta Me}$.

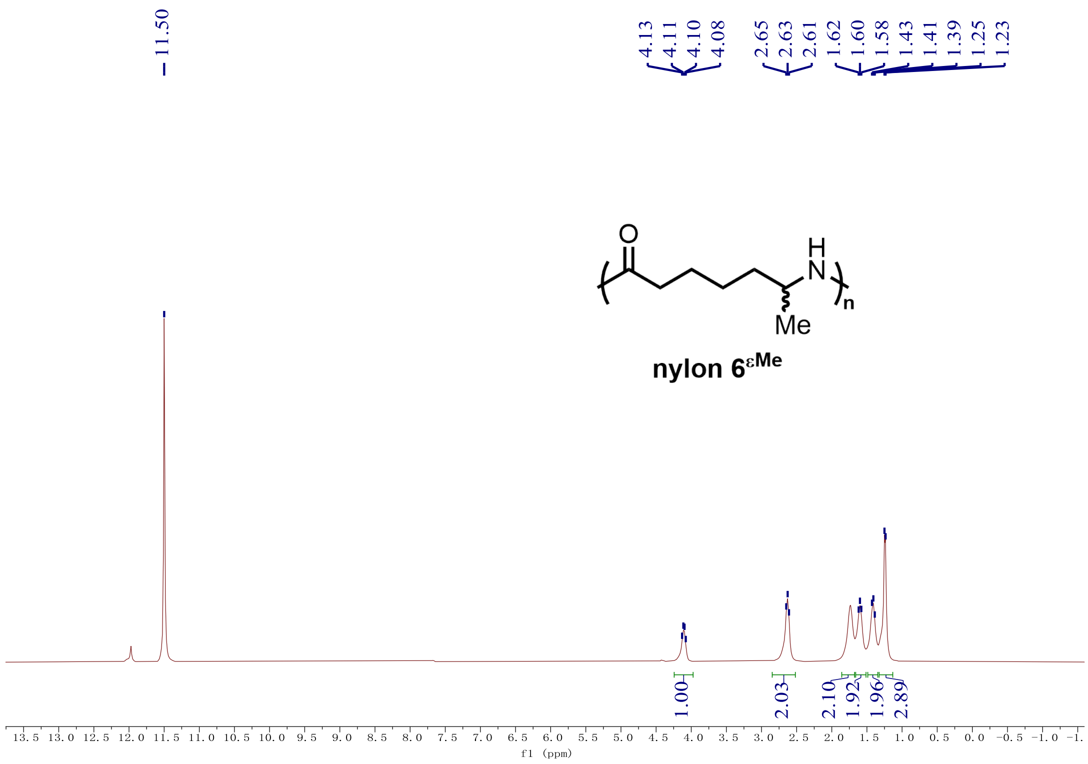

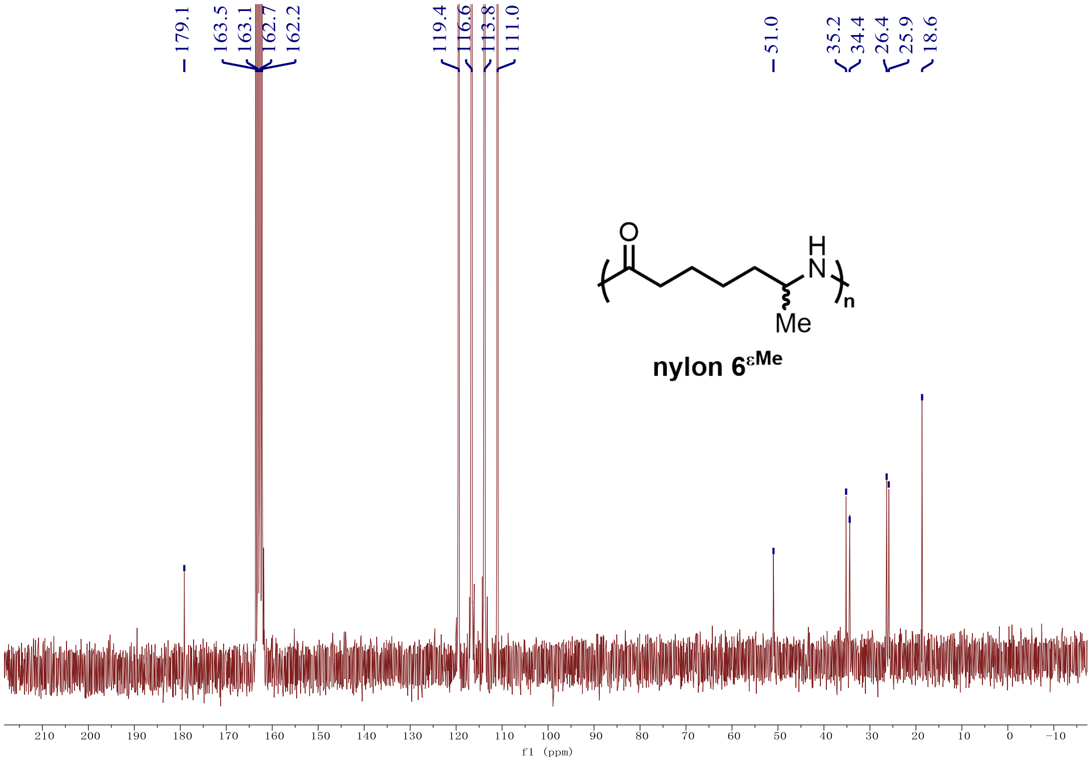

Figure S12. ^{1}H and ^{13}C NMR (TFA-d_1, 25 °C) spectra of nylon $6^{\varepsilon Me}$.

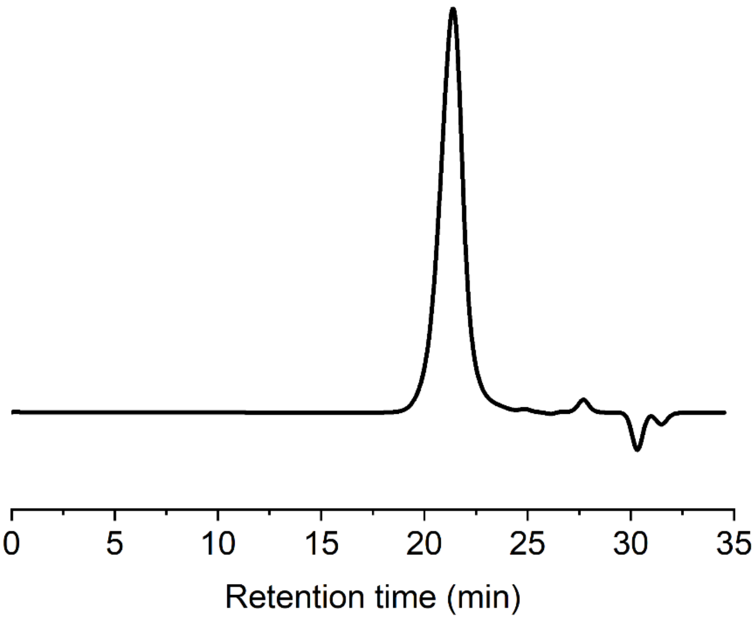

Figure S13. SEC chromatogram of nylon $6^{\alpha Me}$ (M_n = 7.13 kDa, $Đ$ = 1.05) prepared with [$7LM^{\alpha Me}$]/[^{t}Bu-P_4]/[**A1**] = 100:1:1 at 60 °C in 2 M DMAc. System peaks eluted at retention time > 25 min.

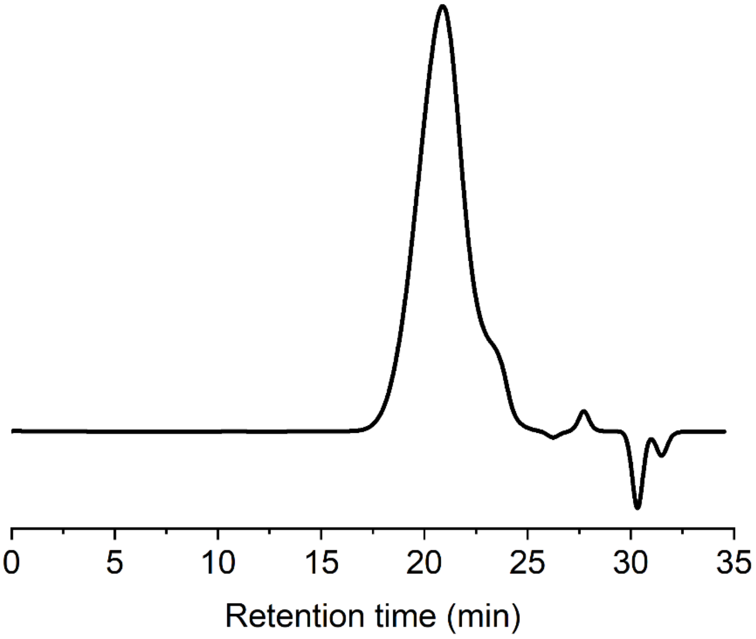

Figure S14. SEC chromatogram of nylon $6^{\alpha Me}$ (M_n = 6.90 kDa, $Đ$ = 1.49) prepared with [$7LM^{\alpha Me}$]/[^{t}Bu-P_4]/[**A1**] = 100:1:1 at 80 °C in 2 M DMAc. System peaks eluted at retention time > 25 min.

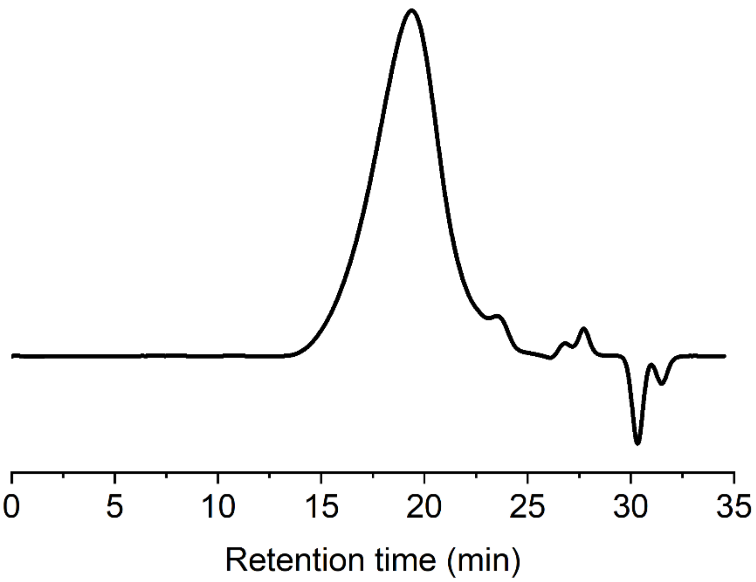

Figure S15. SEC chromatogram of nylon $6^{\alpha Me}$ (M_n = 13.7 kDa, $Đ$ = 2.79) prepared with $[7LM^{\alpha Me}]/[^tBu\text{-}P_4]/[\mathbf{A1}]$ = 100:1:1 at 100 °C in bulk. System peaks eluted at retention time > 25 min.

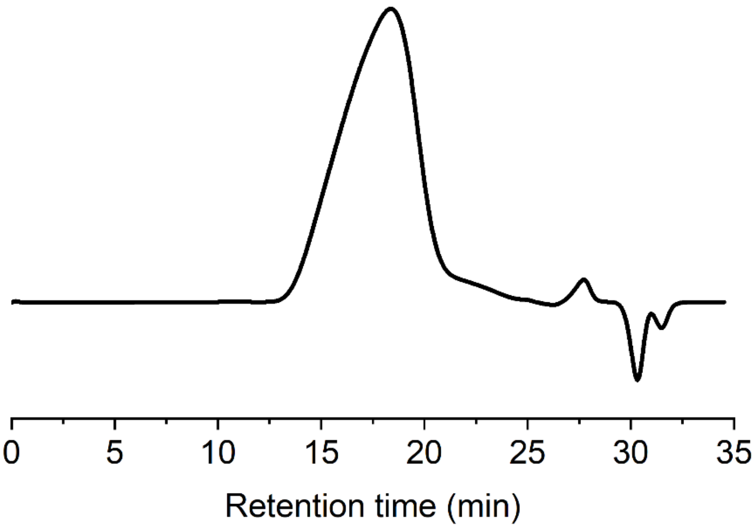

Figure S16. SEC chromatogram of nylon $6^{\alpha Me}$ (M_n = 38.6 kDa, $Đ$ = 2.36) prepared with $[7LM^{\alpha Me}]/[^tBu\text{-}P_4]/[\mathbf{A1}]$ = 500:1:1 at 100 °C in bulk. System peaks eluted at retention time > 25 min.

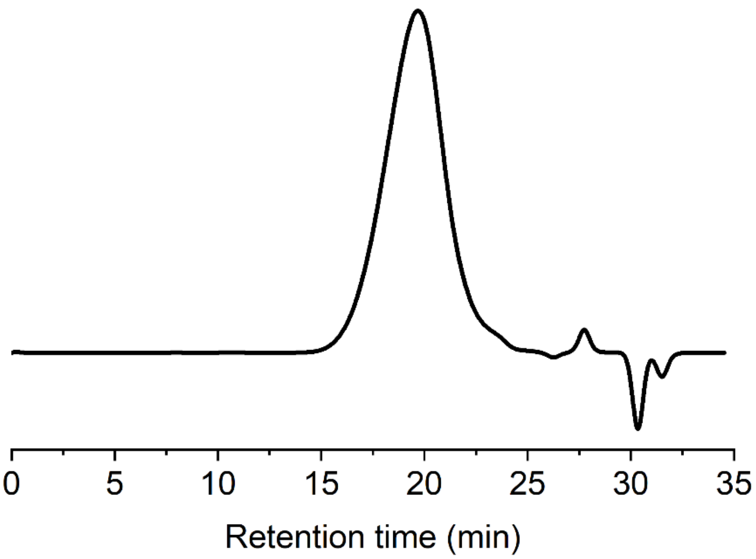

Figure S17. SEC chromatogram of nylon $6^{\beta Me}$ (M_n = 14.6 kDa, $Đ$ = 1.77) prepared with [$7LM^{\beta Me}$]/[^{t}Bu-P_4]/[**A1**] = 100:1:1 at 100 °C in bulk. System peaks eluted at retention time > 25 min.

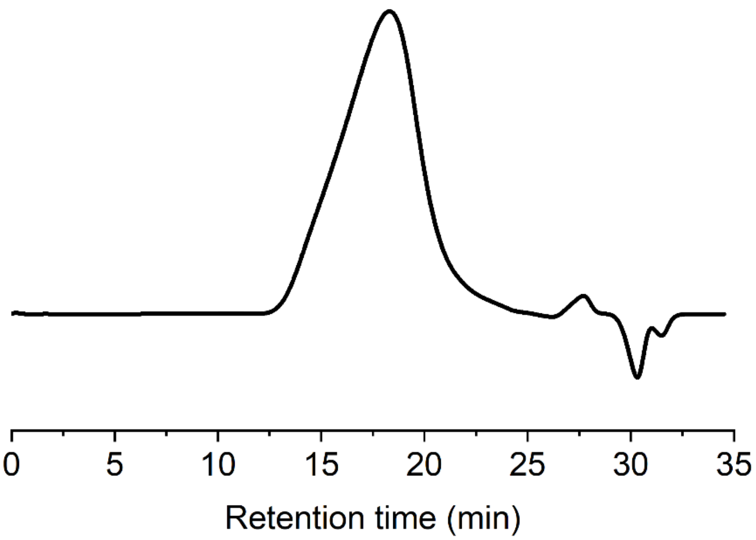

Figure S18. SEC chromatogram of nylon $6^{\beta Me}$ (M_n = 36.1 kDa, $Đ$ = 2.54) prepared with [$7LM^{\beta Me}$]/[^{t}Bu-P_4]/[**A1**] = 500:1:1 at 100 °C in bulk. System peaks eluted at retention time > 25 min.

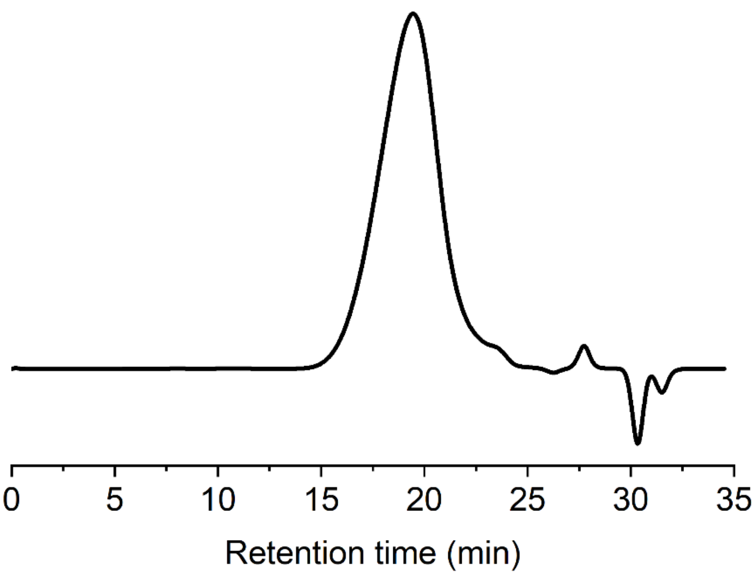

Figure S19. SEC chromatogram of iso-rich nylon $6^{\beta Me}$ (M_n = 15.5 kDa, $Đ$ = 1.88) prepared with [(*R*)-$7LM^{\beta Me}$]/[$7LM^{\beta Me}$]/[^{t}Bu-P_4]/[**A1**] = 50:50:1:1 at 100 °C in bulk. System peaks eluted at retention time > 25 min.

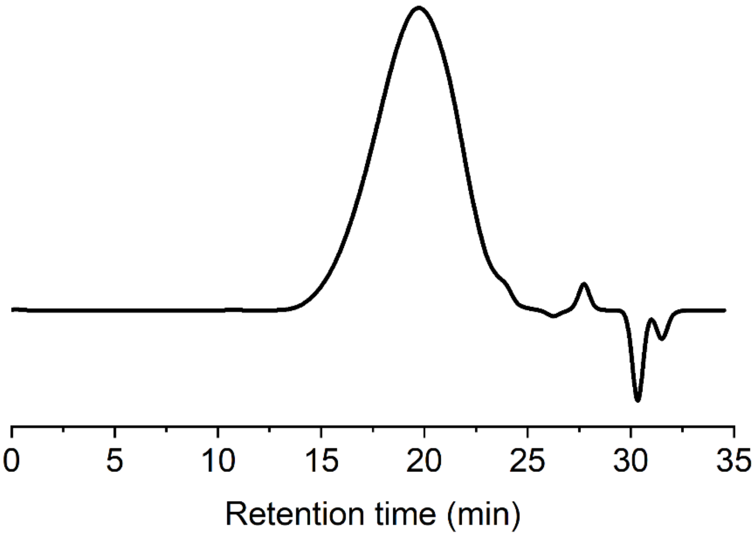

Figure S20. SEC chromatogram of isotactic nylon $6^{\beta Me}$ (M_n = 11.8 kDa, $Đ$ = 2.61) prepared with [(*R*)-$7LM^{\beta Me}$]/[^{t}Bu-P_4]/[**A1**] = 100:1:1 at 100 °C in bulk. System peaks eluted at retention time > 25 min.

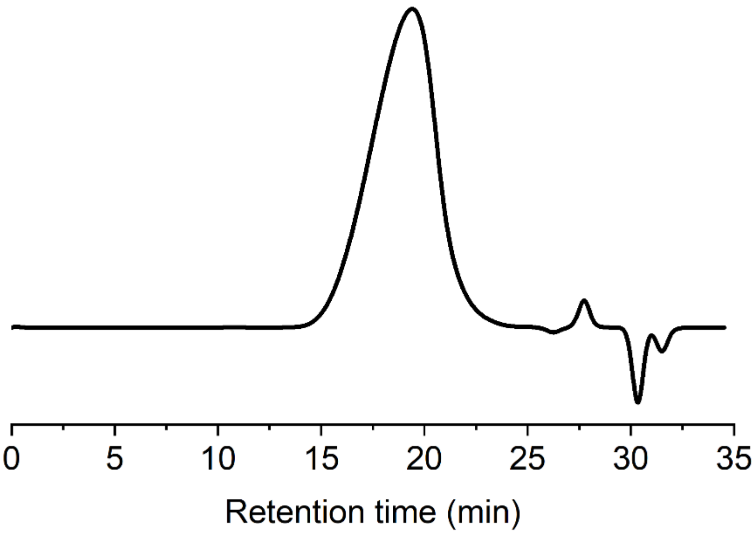

Figure S21. SEC chromatogram of nylon $6^{\delta Me}$ (M_n = 21.0 kDa, $Đ$ = 1.76) prepared with [$7LM^{\delta Me}$]/[^{t}Bu-P_4]/[**A1**] = 100:1:1 at 80 °C in bulk. System peaks eluted at retention time > 25 min.

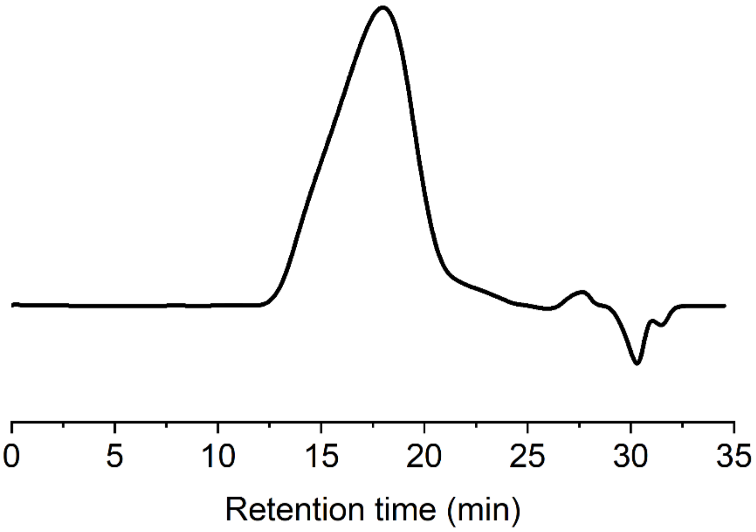

Figure S22. SEC chromatogram of nylon $6^{\delta Me}$ (M_n = 55.7 kDa, $Đ$ = 2.18) prepared with [$7LM^{\delta Me}$]/[^{t}Bu-P_4]/[**A1**] = 500:1:1 at 80 °C in bulk. System peaks eluted at retention time > 25 min.

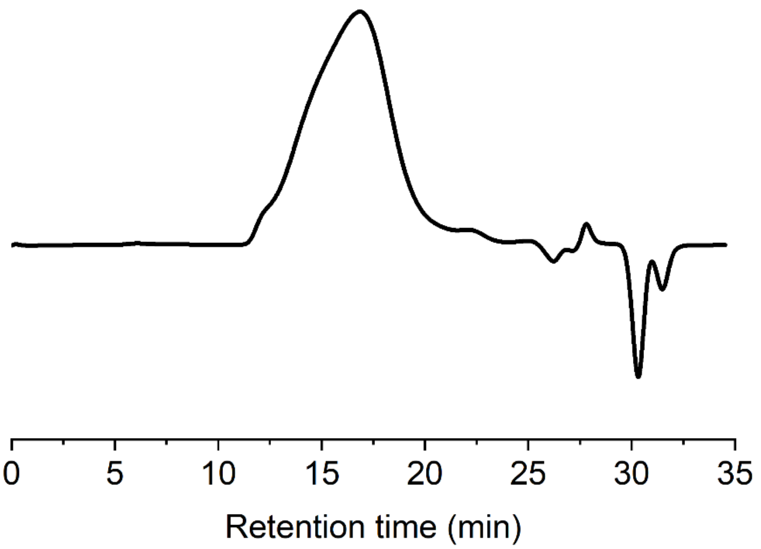

Figure S23. SEC chromatogram of nylon $6^{\varepsilon Me}$ (M_n = 34.4 kDa, $Đ$ = 3.26) prepared with [$7LM^{\varepsilon Me}$]/[NaH]/[**A1**] = 100:1:1 at 140 °C in bulk. System peaks eluted at retention time > 25 min.

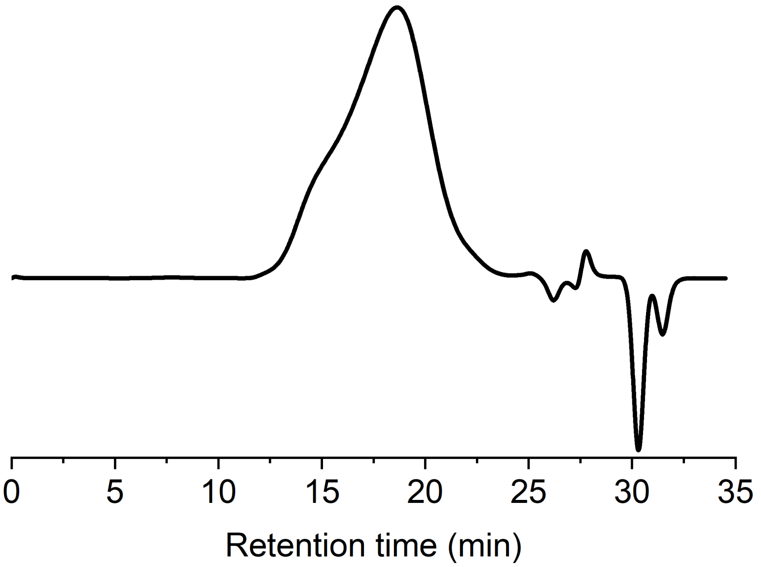

Figure S24. SEC chromatogram of nylon $6^{\varepsilon Me}$ (M_n = 103 kDa, $Đ$ = 2.55) prepared with [$7LM^{\varepsilon Me}$]/[NaH]/[**A1**] = 500:1:1 at 140 °C in bulk. System peaks eluted at retention time > 25 min.

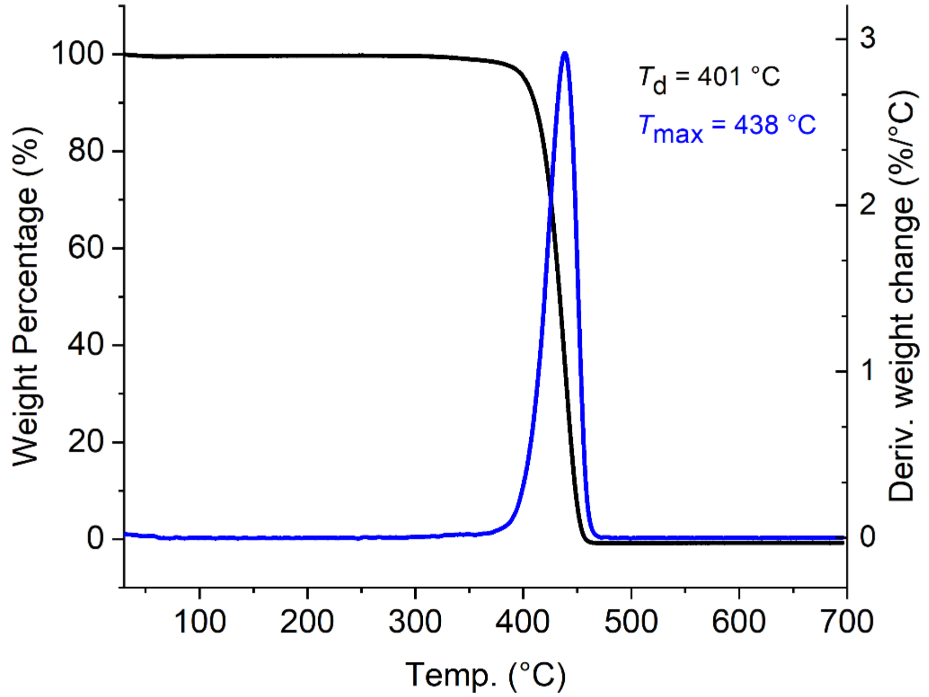

Figure S25. TGA and DTG curves of nylon $6^{\alpha Me}$ (M_n = 6.90 kDa, $Đ$ = 1.49).

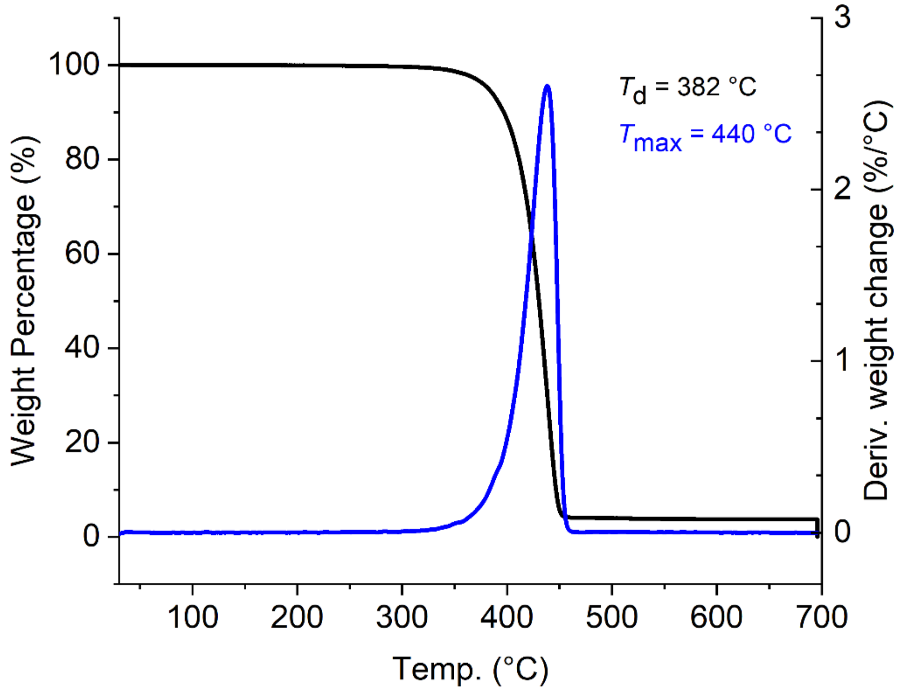

Figure S26. TGA and DTG curves of nylon $6^{\beta Me}$ (M_n = 14.6 kDa, $Đ$ = 1.77).

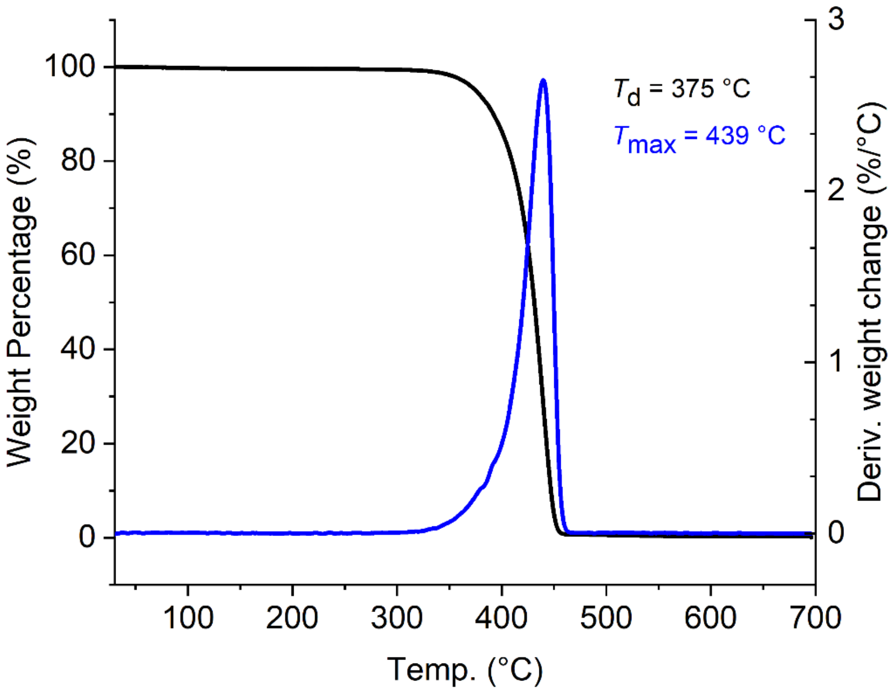

Figure S27. TGA and DTG curves of nylon $6^{\delta Me}$ (M_n = 21.0 kDa, $Đ$ = 1.76).

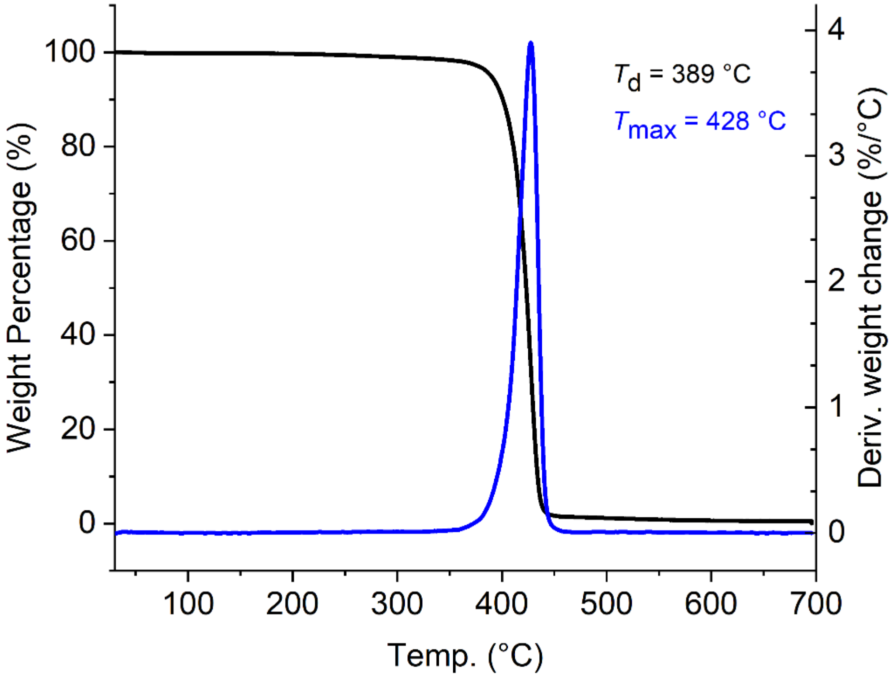

Figure S28. TGA and DTG curves of nylon $6^{\varepsilon Me}$ (M_n = 34.4 kDa, $Đ$ = 3.26).

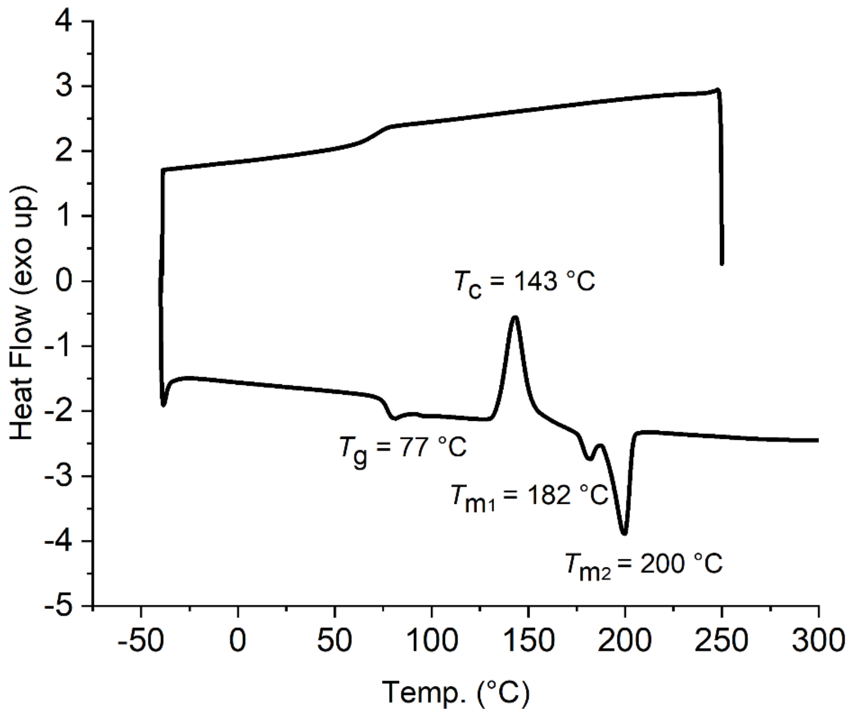

Figure S29. DSC curves of 1st cooling and 2nd heating scans of nylon $6^{\alpha Me}$ (M_n = 6.90 kDa, $Đ$ = 1.49). Scan rate: 10 °C/min.

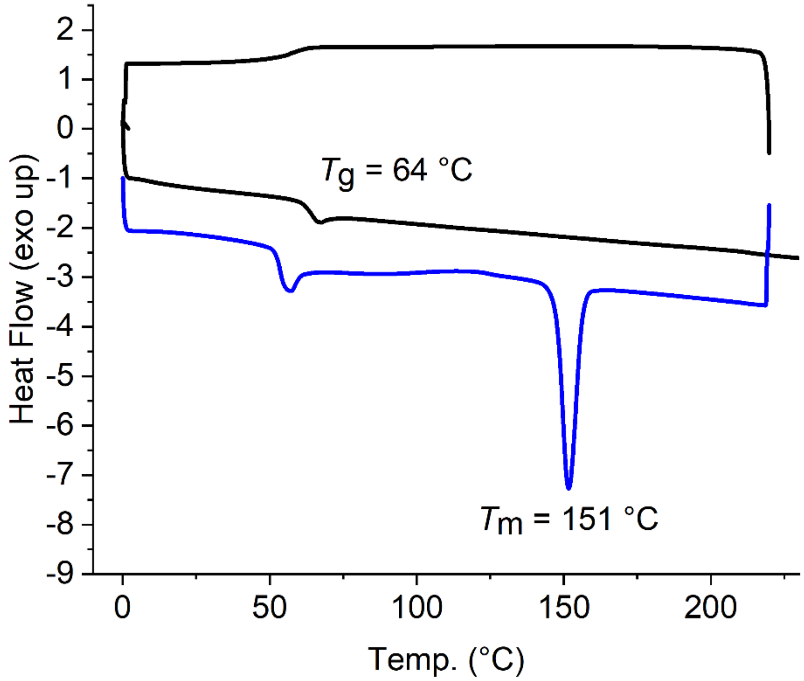

Figure S30. DSC curves of 1st heating (blue line), cooling scans and 2nd heating scan (black line) of nylon $6^{\beta Me}$ (M_n = 14.6 kDa, $Đ$ = 1.77). Scan rate: 10 °C/min.

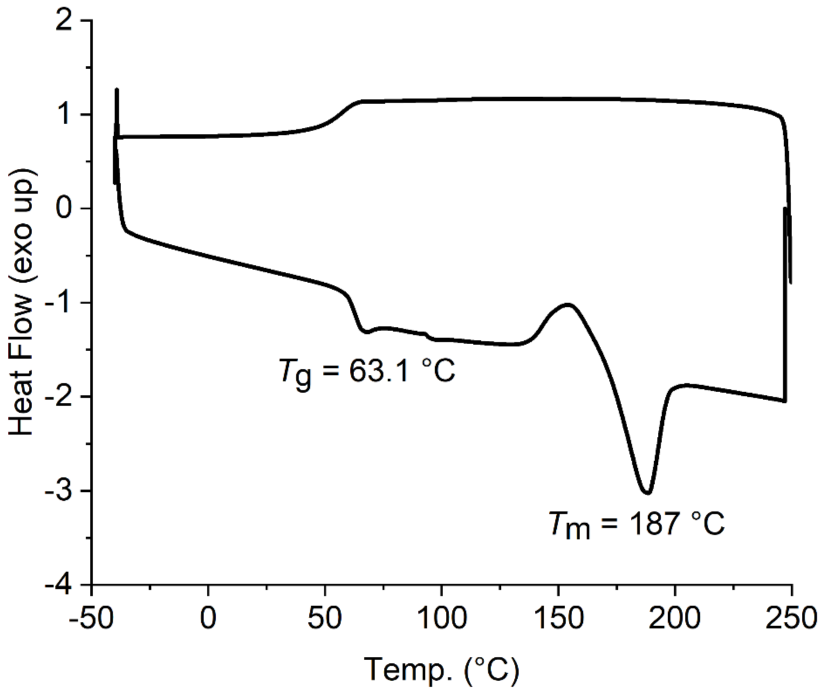

Figure S31. DSC curves of 1st cooling and 2nd heating scans of iso-rich nylon $6^{\beta Me}$ (M_n = 15.5 kDa, $Đ$ = 1.88). Scan rate: 10 °C/min.

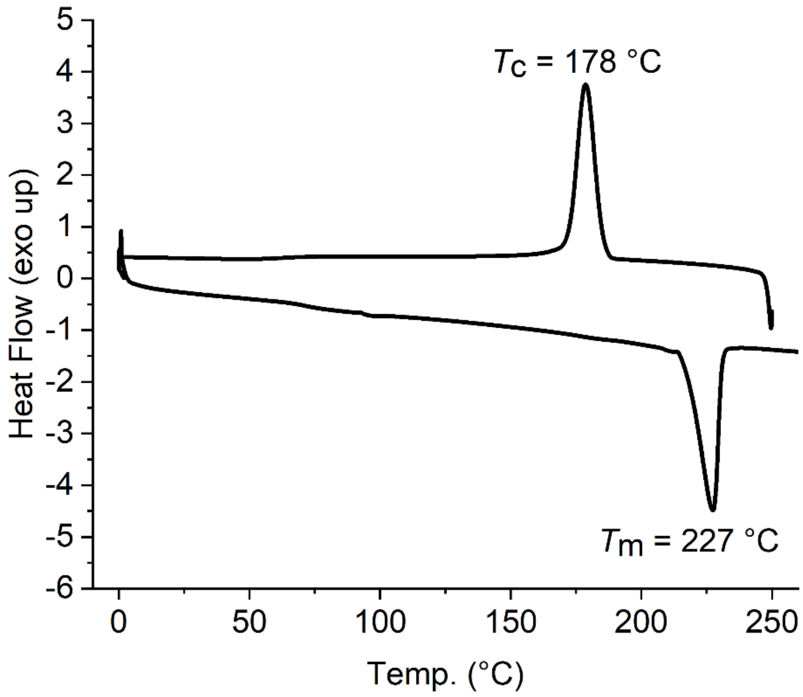

Figure S32. DSC curves of 1st cooling and 2nd heating scans of isotactic nylon $6^{\beta Me}$ (M_n = 11.8 kDa, $Đ$ = 2.61). Scan rate: 10 °C/min.

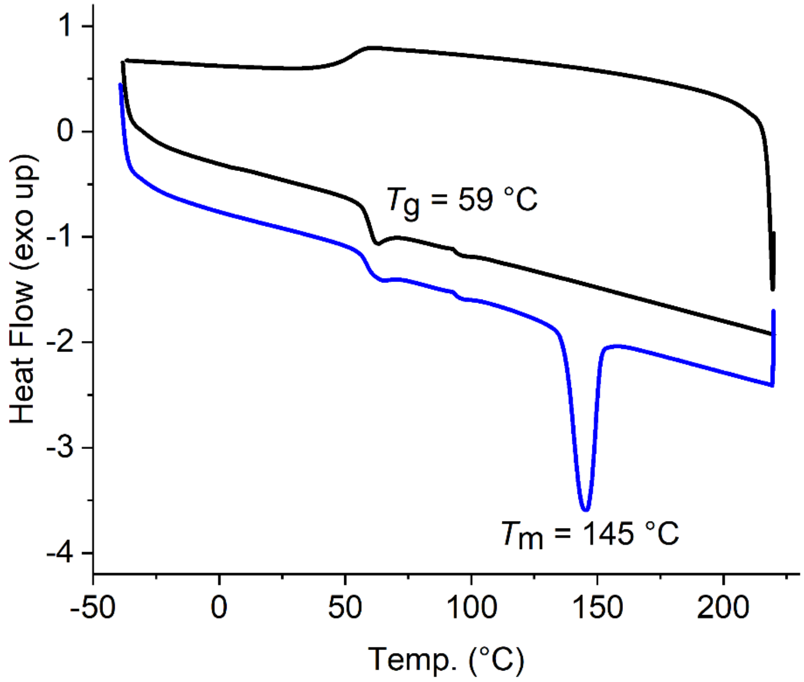

Figure S33. DSC curves of 1st heating (blue line) and cooling scans (black line) and 2nd heating scan (black line) of nylon $6^{\delta Me}$ (M_n = 21.0 kDa, Đ = 1.76). Scan rate: 10 °C/min.

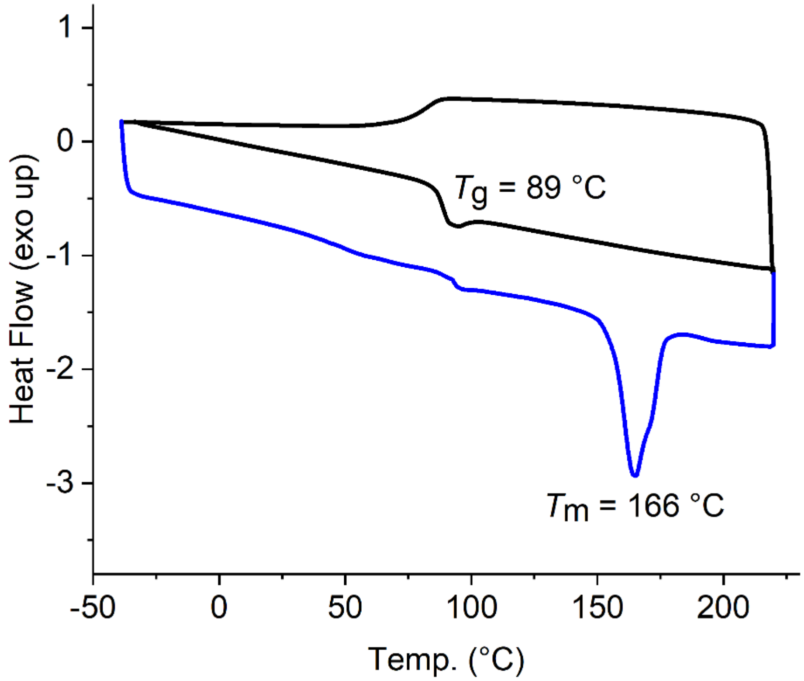

Figure S34. DSC curves of 1st heating (blue line) and cooling scans (black line) and 2nd heating scan (black line) of nylon $6^{\varepsilon Me}$ (M_n = 34.4 kDa, Đ = 3.26). Scan rate: 10 °C/min.

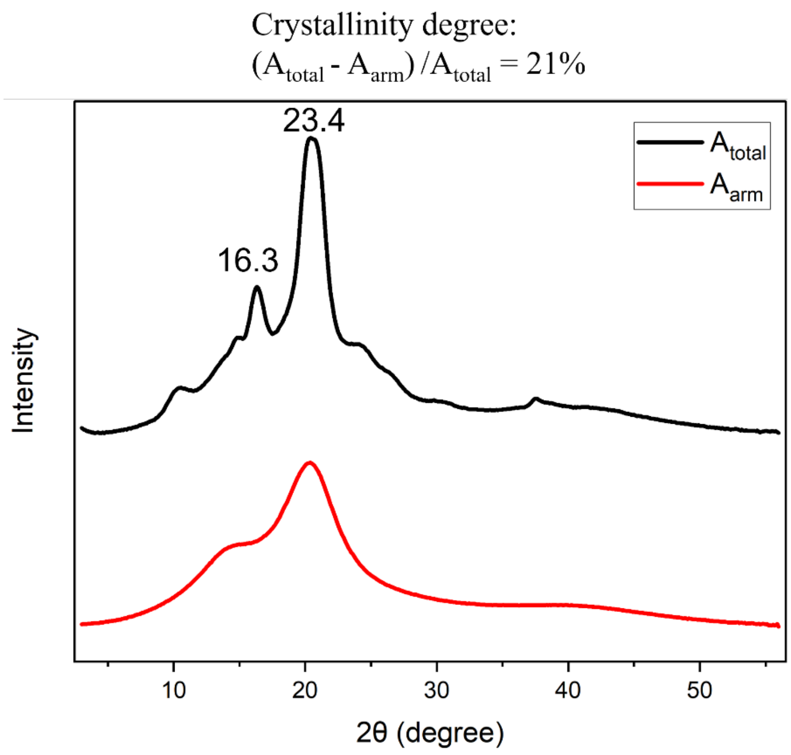

Figure S35. WAXS profiles of semicrystalline and amorphous nylon $6^{\alpha Me}$.

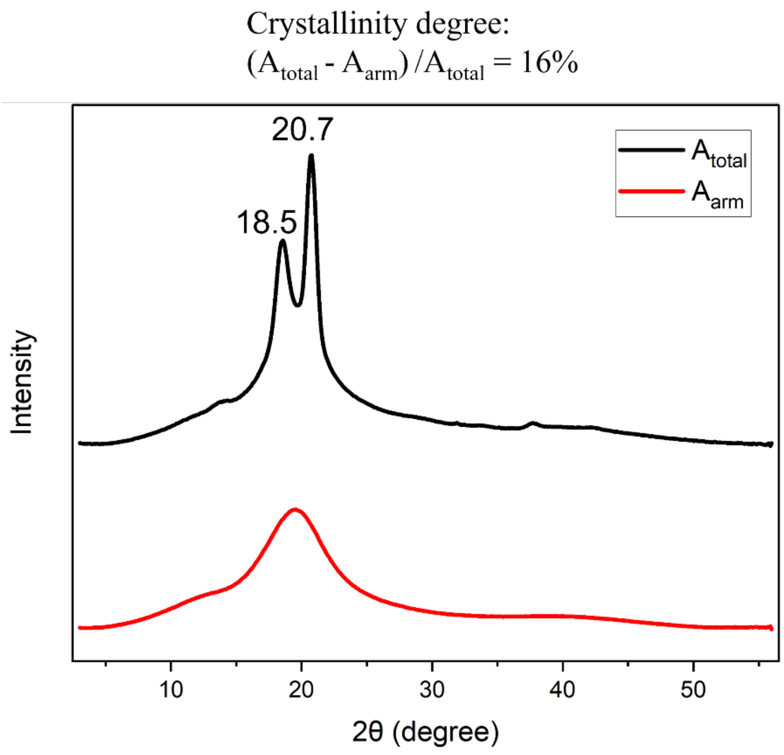

Figure S36. WAXS profiles of semicrystalline and amorphous nylon $6^{\beta Me}$.

Crystallinity degree:
$(A_{total} - A_{arm}) / A_{total} = 12\%$

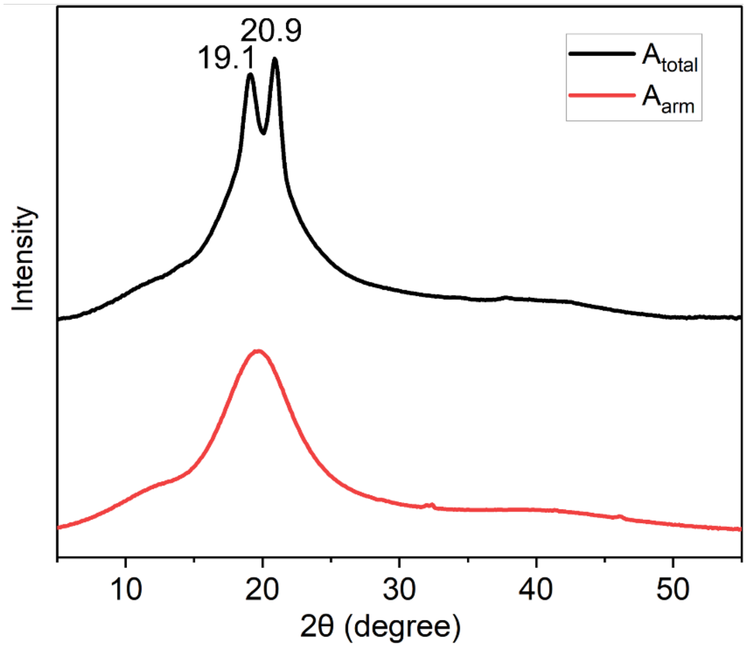

Figure S37. WAXS profiles of semicrystalline and amorphous nylon $6^{\delta Me}$.

Crystallinity degree:
$(A_{total} - A_{arm}) / A_{total} = 17\%$

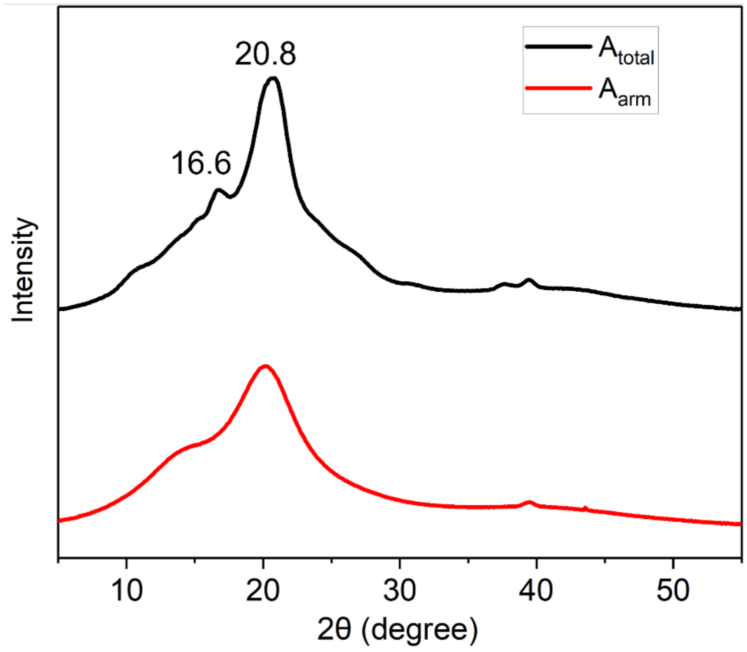

Figure S38. WAXS profiles of semicrystalline and amorphous nylon $6^{\varepsilon Me}$.

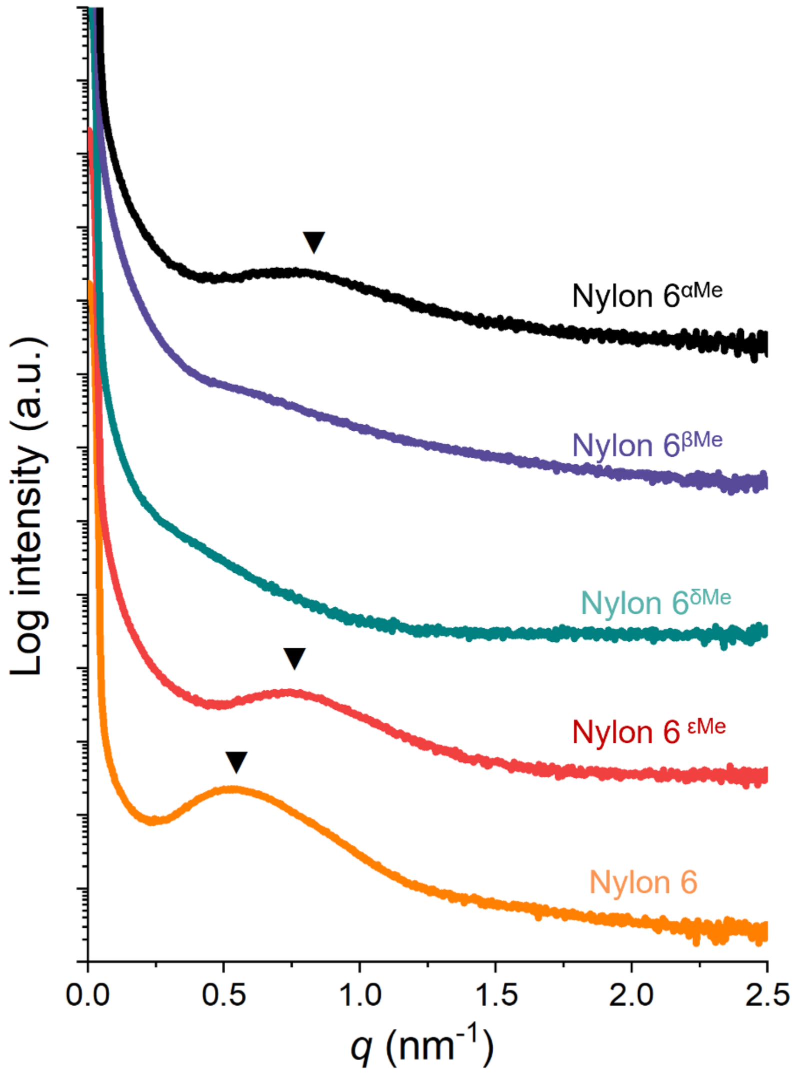

Figure S39. Small-angle X-ray scattering (SAXS) of methyl-substituted nylon-6 variants and nylon 6.

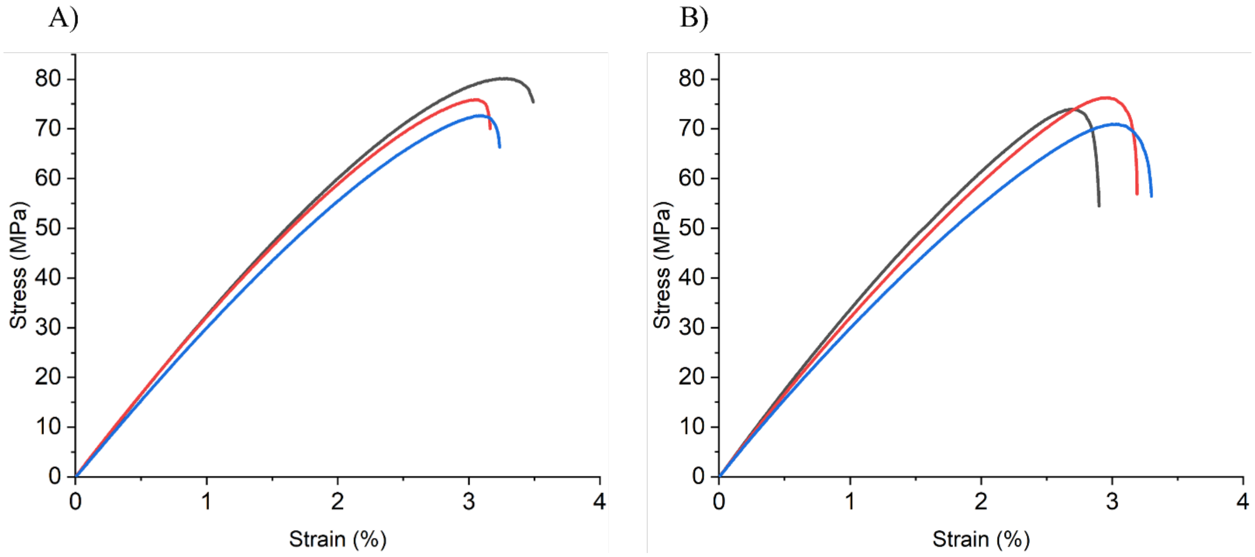

Figure S40. Tensile curves: A) nylon $6^{\alpha Me}$ (M_n = 38.6 kDa, $Đ$ = 2.36); B) nylon $6^{\alpha Me}$ (M_n = 87.0 kDa, $Đ$ = 2.14), strain rate = 5 mm/min, ambient conditions.

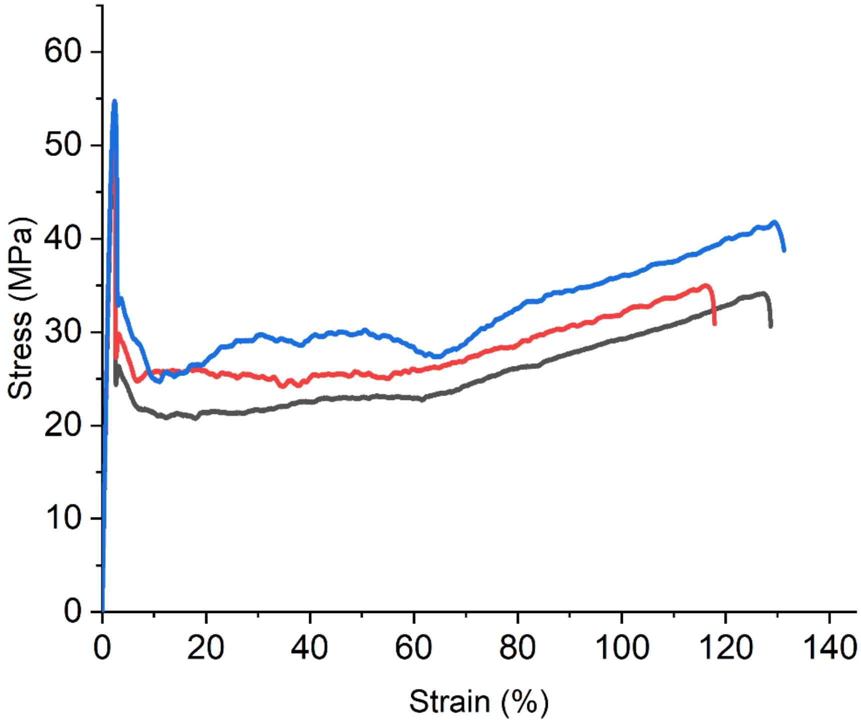

Figure S41. Tensile curves for nylon $6^{\beta Me}$ (M_n = 36.1 kDa, $Đ$ = 2.54), strain rate = 5 mm/min, ambient condition.

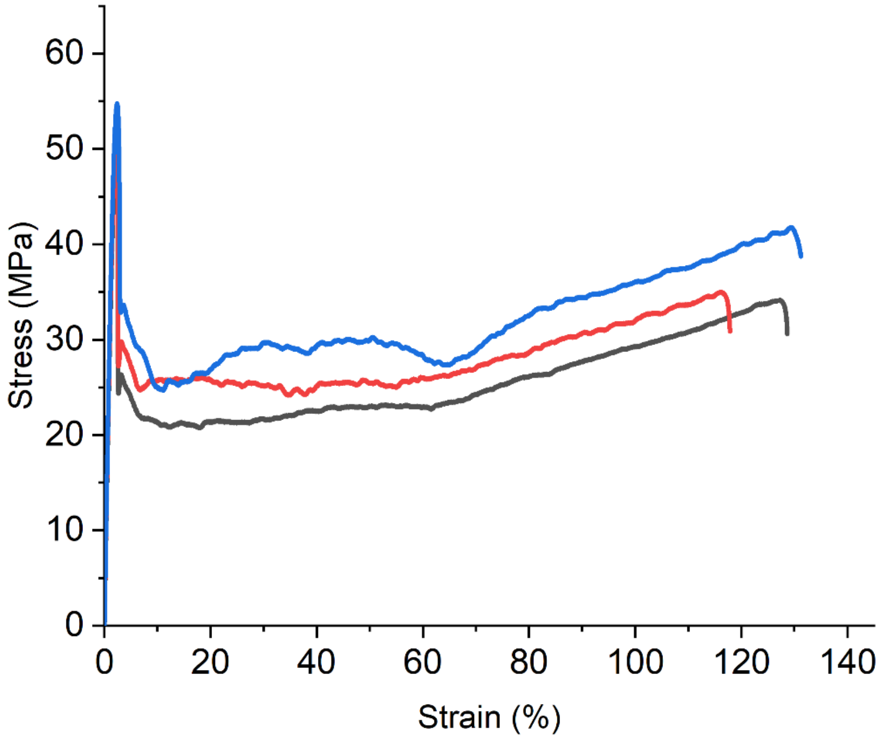

Figure S42. Tensile curves for nylon 6$^{\delta Me}$ (M_n = 55.7 kDa, $Đ$ = 2.18), strain rate = 5 mm/min, ambient condition.

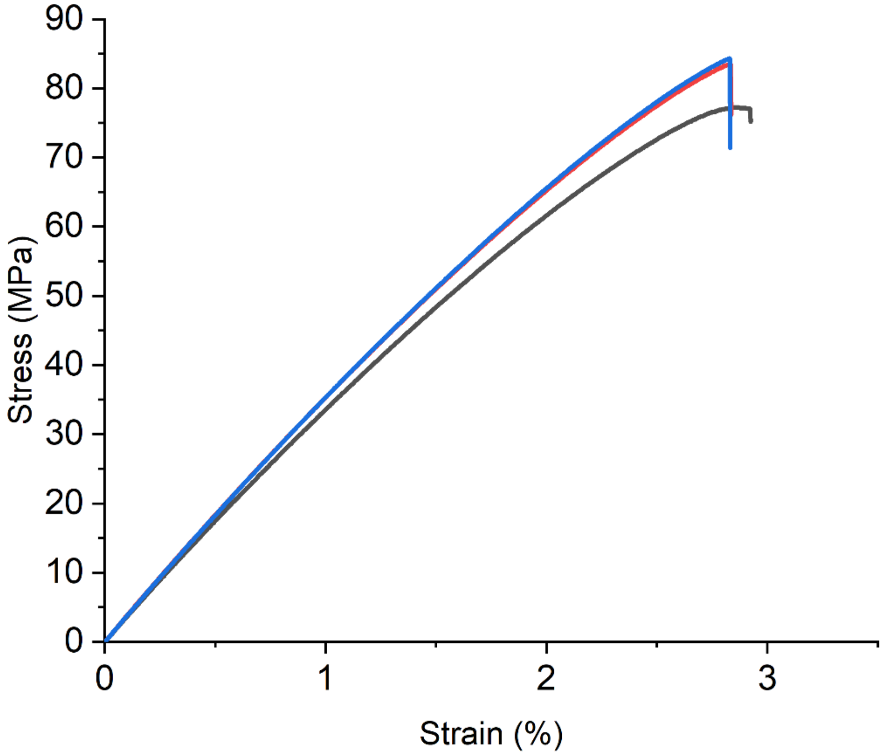

Figure S43. Tensile curves for nylon 6$^{\varepsilon Me}$ (M_n = 103 kDa, $Đ$ = 2.55), strain rate = 5 mm/min, ambient condition.

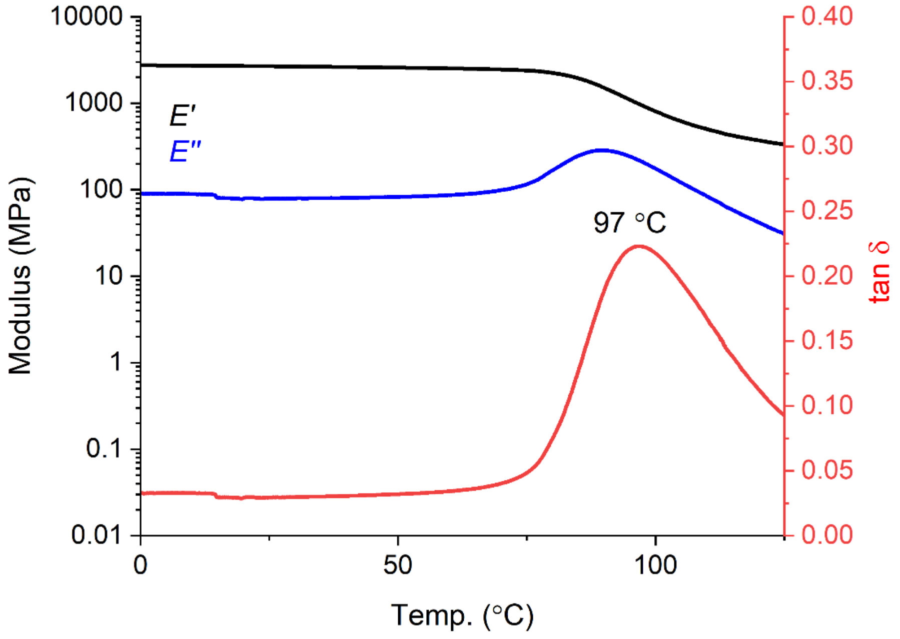

Figure S44. Overlay plots of storage modulus E', loss modulus E'', and tan δ (E''/E') for nylon $6^{\alpha Me}$ (M_n = 38.6 kDa, $Đ$ = 2.36) measured by DMA (tension film mode, 0.1% strain, 1 Hz, 3 °C per min).

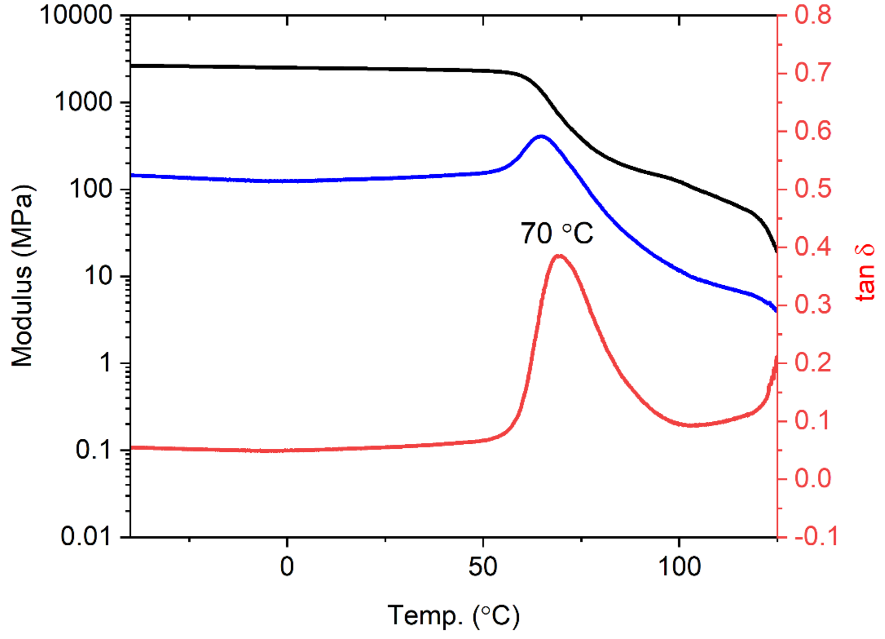

Figure S45. Overlay plots of storage modulus E', loss modulus E'', and tan δ (E''/E') for nylon $6^{\beta Me}$ (M_n = 36.1 kDa, $Đ$ = 2.54) measured by DMA (tension film mode, 0.1% strain, 1 Hz, 3 °C per min).

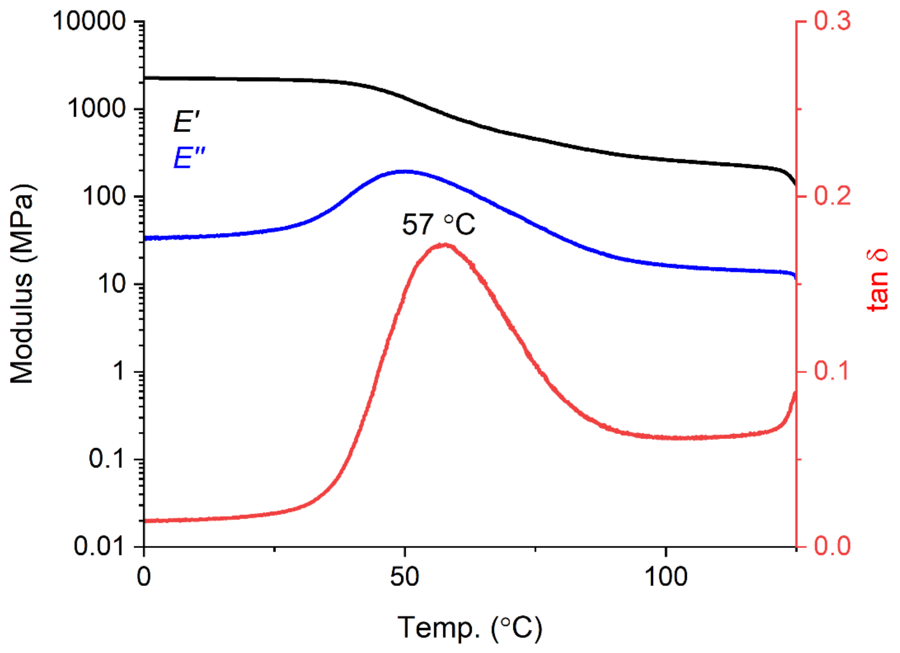

Figure S46. Overlay plots of storage modulus E', loss modulus E'', and tan δ (E''/E') for nylon $6^{\delta Me}$ (M_n = 55.7 kDa, $Đ$ = 2.18) measured by DMA (tension film mode, 0.1% strain, 1 Hz, 3 °C per min).

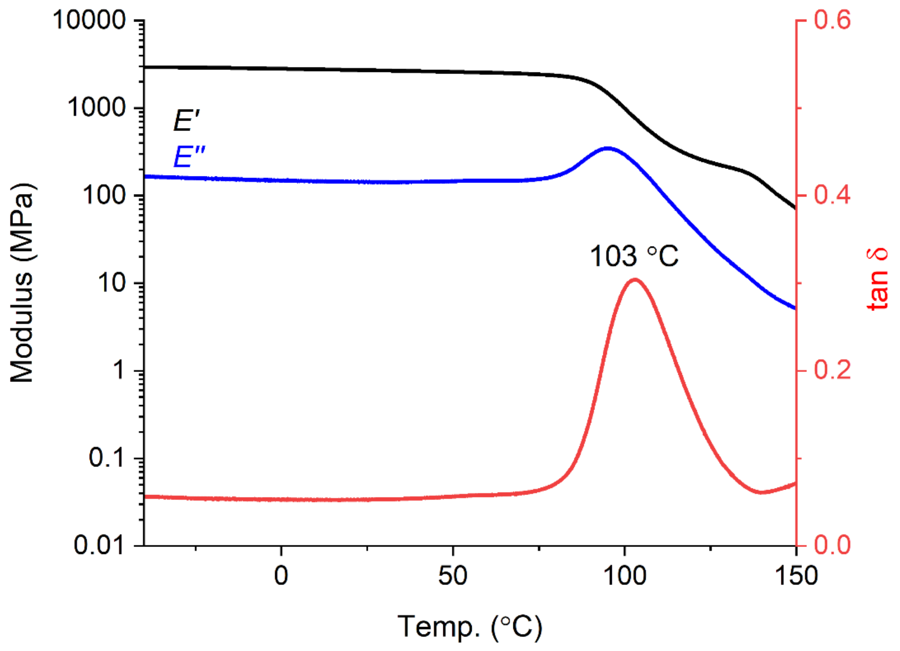

Figure S47. Overlay plots of storage modulus E', loss modulus E'', and tan δ (E''/E') for nylon $6^{\varepsilon Me}$ (M_n = 103 kDa, $Đ$ = 2.55) measured by DMA (tension film mode, 0.1% strain, 1 Hz, 3 °C per min).

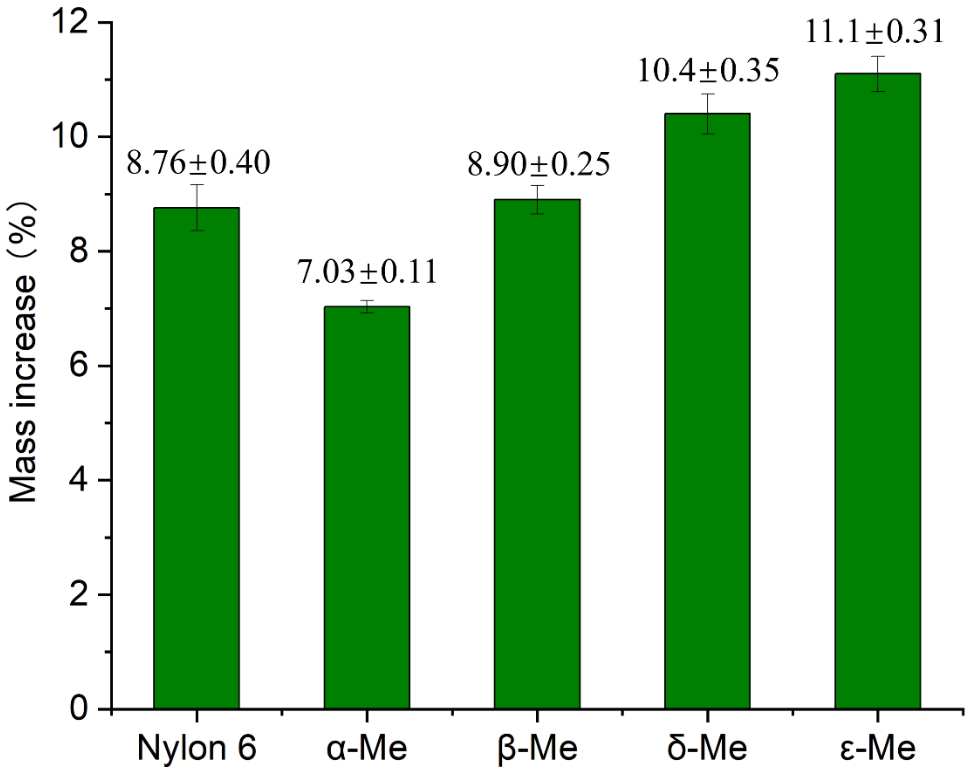

Figure S48. Water absorption after 7 days of immersion.

Supplementary Tables

Table S1. Polymerization of (*R*)-7LM$^{\beta Me}$ and 7LM$^{\beta Me}$

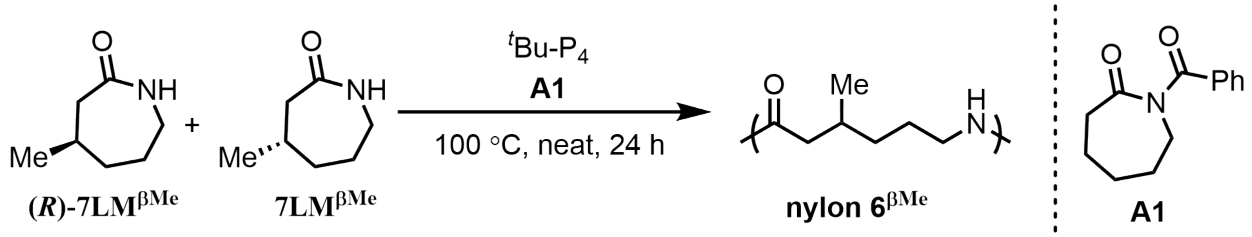

Run[a]	[(*R*)-M]/[M]/[B]/[**A1**]	Tacticity	Yield (%)[b]	M_n (kDa)[c]	$Đ$[c]
1	100/0/1/1	isotactic	75	11.8	2.61
2	50/50/1/1	iso-rich	73	15.5	1.88
3	0/100/1/1	atactic	90	14.6	1.77

[a] Conditions: Monomer (M = 7LM$^{\beta Me}$) (2 mmol), Base (B) = ^{t}Bu-P$_4$, activator = **A1**. [b] Isolated yield. [c] Number-average molar mass (M_n) and dispersity index ($Đ$ = M_w/M_n) determined via size exclusion chromatography (SEC) coupled with a Wyatt Technology miniDAWN TREOS Multi-Angle Light scattering detector and a Wyatt Technology Optilab T-rEX differential refractometer.

Table S2. Results of the depolymerization of methyl nylon-6 variants

Run[a]	Polymer	Catalyst	T/°C[b]	t/h	Yield/%[c]
1	nylon 6$^{\alpha Me}$	^{t}BuOK (10 wt%)	260	12	81
2	nylon 6$^{\beta Me}$	^{t}BuOK (10 wt%)	250	6	92
3	nylon 6$^{\delta Me}$	^{t}BuOK (10 wt%)	260	24	82
4	nylon 6$^{\varepsilon Me}$	^{t}BuOK (10 wt%)	260	24	77

[a] Conditions: polymer (254 mg, 2 mmol), under 200 mTorr. [b] Temperature of the thermocouple-controlled heating mantle. [c] Isolated yield.

Table S3. Polymerization of recovered 7LM^{Me}[a]

Run	recovered 7LM^{Me}	[M]/[B]/[A]	T (°C)	Base	Yield[b] (%)	M_n (kDa)[c]	$Đ$[c]
1	7LM$^{\alpha Me}$	100/1/1	100	^{t}Bu-P$_4$	88	13.5	2.35

2	$7LM^{\beta Me}$	100/1/1	100	^{t}Bu-P_4	89	15.0	1.83
3	$7LM^{\delta Me}$	100/1/1	80	^{t}Bu-P_4	84	11.5	3.13
4	$7LM^{\varepsilon Me}$	100/1/1	140	NaH	71	34.3	2.82

[a] Conditions: Monomer (M) (1 mmol), Base (B), activator = **A1,** neat, 12 hours. [b] Isolated yield. [c] Number-average molar mass (M_n) and dispersity index ($Đ = M_w/M_n$) determined via size exclusion chromatography (SEC) coupled with a Wyatt Technology miniDAWN Multi-Angle Light scattering detector and a Wyatt Technology Optilab differential refractometer.

Table S4. Tensile stress-strain data of nylon $6^{\alpha Me}$ (M_n = 38.6 kDa, $Đ$ = 2.36) dog-bone-shaped specimens

Entry	Young's modulus (MPa)	elongation at break (%)	yield stress (MPa)	ultimate tensile strength (MPa)
1	3350	3.5	80.1	75.3
2	3340	3.2	75.8	70.0
3	3090	3.2	72.6	66.3
Average	3260	3.3	76.1	70.5
Standard deviation	147	0.2	3.8	4.5

Table S5. Tensile stress-strain data of nylon $6^{\beta Me}$ (M_n = 36.1 kDa, $Đ$ = 2.54) dog-bone-shaped specimens

Entry	Young's modulus (MPa)	elongation at break (%)	yield stress (MPa)	ultimate tensile strength (MPa)
1	3220	96.6	49.0	29.6
2	3270	92.5	50.1	35.6
3	3320	106	50.4	32.4
Average	3270	98.3	49.8	32.5
Standard deviation	50	6.9	0.7	3.0

Table S6. Tensile stress-strain data of nylon $6^{\delta Me}$ (M_n = 55.7 kDa, $Đ$ = 2.18) dog-bone-shaped specimens

Entry	Young's modulus (MPa)	elongation at break (%)	yield stress (MPa)	ultimate tensile strength (MPa)
1	3230	127	49.2	34.1
2	3300	117	53.0	34.8
3	3370	129	54.8	41.7
Average	3300	124	52.3	36.8
Standard deviation	70	6.4	2.8	4.2

Table S7. Tensile stress-strain data of nylon $6^{\varepsilon Me}$ (M_n = 103 kDa, $Đ$ = 2.55) dog-bone-shaped specimens

Entry	Young's modulus (MPa)	elongation at break (%)	yield stress (MPa)	ultimate tensile strength (MPa)
1	3510	2.9	77.2	77.2
2	3680	2.8	83.5	83.5
3	3670	2.8	84.3	84.3
Average	3620	2.8	81.6	81.6
Standard deviation	95	0.05	3.9	3.9

References

[1] J. J. Tian, X. Liu, L. Ye, Z. Zhang, E. C. Quinn, C. Shi, Linda J. Broadbelt, T. J. Marks, E. Y.-X. Chen, *Angew. Chem. Int. Ed.* **2024**, *63*, e202320214.

[2] C. G. Overberger, H. Jabloner, *J. Am. Chem. Soc.* **1963**, *85*, 3431–3435.
